# Breast Cancer Primary Prevention and Diet: An Umbrella Review

**DOI:** 10.3390/ijerph17134731

**Published:** 2020-07-01

**Authors:** Alessandra Buja, Marco Pierbon, Laura Lago, Giulia Grotto, Vincenzo Baldo

**Affiliations:** 1 Department of Cardiological, Thoracic and Vascular Sciences, University of Padova, Via Loredan 18, 35127 Padova, Italy; giulia.grotto.1@studenti.unipd.it (G.G.); vincenzo.baldo@unipd.it (V.B.); 2 Masters Course in Sciences for the Public Health and Prevention Professions, University of Padova, Via Giustiniani 2, 35127 Padova, Italy; marco.pierbon94@gmail.com (M.P.); laura.lago@unipd.it (L.L.)

**Keywords:** diet, cancer risk, breast cancer, cancer prevention, dietary intervention

## Abstract

*Introduction:* Many studies have been published, but none have pooled the useful evidence available in the literature to produce guidelines and health policies promoting healthy eating styles to prevent breast cancer (BC). The present study aimed to summarize the evidence produced to date, taking a judicious, critical approach to the quality of the studies analyzed. *Methods:* An umbrella review method was adopted, which is a systematic review of second-level studies, meta-analyses and literature reviews. *Results:* In all, 48 studies were considered: 32 meta-analyses, 4 pooled analyses, 5 systematic reviews, and 7 qualitative reviews. A higher intake of total meat, or red or processed meats, or foods with a high glycemic index, or eggs would seem to be associated with a higher risk of BC. Some foods, such as vegetables, would seem instead to have an inverse association with BC risk. One meta-analysis revealed an inverse association between citrus fruit and mushroom consumption and BC. Some nutrients, such as calcium, folate, vitamin D, lignans and carotenoids, also seem to be inversely associated with BC risk. The evidence is still conflicting as concerns exposure to other dietary elements (e.g., polyunsaturated fatty acids, dairy foods). *Conclusion:* Nutrition is one of the most modifiable aspects of people’s lifestyles and dietary choices can affect health and the risk of cancer. Overall, adhering to a healthy eating style may be associated with a significant reduction in the risk of BC.

## 1. Introduction

Breast cancer (BC) is the most frequently diagnosed female cancer, accounting for 29% of cancers in women [1]. It is important for primary prevention to include reducing modifiable risk factors, such as obesity, a sedentary lifestyle, and a poor diet. Each of these factors can have various effects, depending on breast tissue type and age (premenopausal and menopausal).

As concerns diet, the role of alcohol as a risk factor has been well established: the results of the 2011 EPIC (European Prospective Investigation into Cancer and Nutrition) survey found that 5% of BCs could be attributable to alcohol consumption [2]. Numerous studies have addressed the role of several other foods too, sometimes reporting divergent results. For example, according to the EPIC survey, a diet rich in saturated fat is associated with a higher risk of estrogen- and progesterone-positive cancer, with a significantly higher hazard ratio [3]. Similar results emerged from a Swedish cohort survey, in which a high dietary fat consumption seemed to lead to a significant increase in the risk of developing BC [4]. On the other hand, a case-control survey conducted in China in 2008 found no significant association between the various types of dietary fat and the odds of cancer [5]. Red meat and animal proteins seem to be associated with an increase in the risk of neoplastic disease: Jansen suggested that consuming them in large amounts anticipates menarche, and this is recognized as a risk factor and predictor of BC [6].

Several epidemiological studies have shown that consuming soy products is associated with a lower incidence of hormone-related tumors, including BC, due to the properties of isoflavones and phytoestrogens [7,8,9].

Despite the many studies conducted on the topic, the evidence in the literature has yet to be pooled and used for the purpose of drawing up guidelines and health policies to promote healthy eating styles. The aim of the present work was thus to obtain a synthesis of the scientific evidence produced over the last 15 years regarding the association between nutrition and BC. The umbrella review approach was adopted, which entails conducting a systematic review of second-level studies, meta-analyses, and literature reviews, and summarizing the evidence available to date in the light of a judicious critical assessment of the quality of the studies involved.

## 2. Materials and Methods

### 2.1. Study Design

An umbrella review was conducted, which involved critically examining the literature, starting from a synthesis of the previously-published second-level research, and performing a critical review of all available evidence [10,11].

### 2.2. Sources

**Figure 1:**
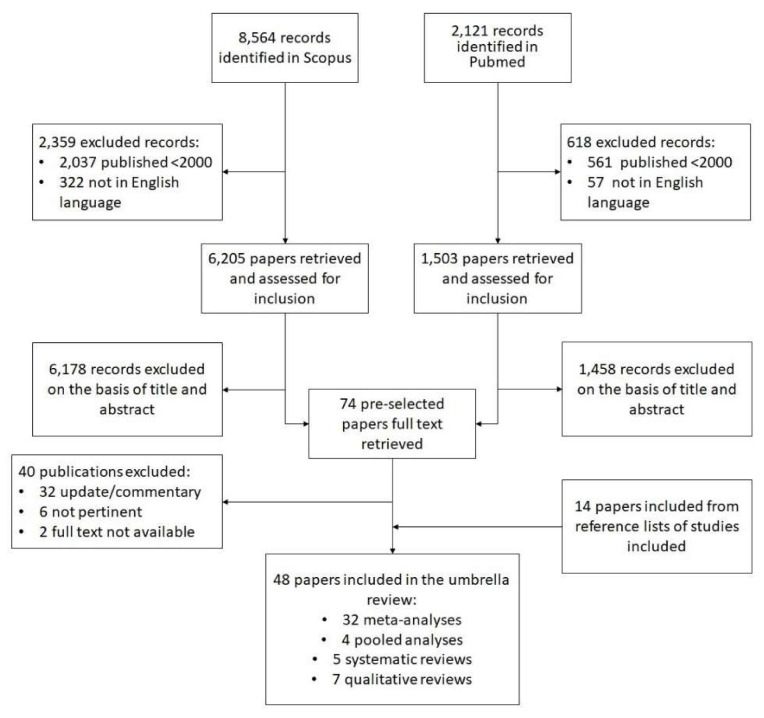
The research strategy used to identify the articles for the umbrella review.

A comprehensive search (see Figure 1) was conducted in the PubMed (Medline) and Scopus databases using combined MeSH terms: (“breast neoplasm” (MeshTerm) OR “breast cancer” OR “breast tumor”) AND (“diet” (MeshTerm) OR “alimentation” OR “nutrition”) AND (“prevention” (MeshTerm) OR “risk” (MeshTerm) OR “association” (MeshTerm)) from 2000 to February 2016. The search concerned meta-analyses, pooled analyses, systematic reviews, and qualitative reviews on the risk of BC in the female population. We checked the references for the systematic reviews and meta-analyses retrieved, and the proceedings of relevant conferences for articles missed by the electronic search. In particular, we included studies that examined the presence and intake of foods in the diet (exogenous exposure), instead of just measuring endogenous nutrients. Studies with the subsequent characteristics were excluded:

1. General and descriptive reports, comments and updates without any reported association measures;
2. Studies on populations or groups at increased risk;
3. Analyses of dietary supplements or studies examining the combined effect of physical activity and diet;
4. Studies involving BC recurrence;
5. Studies written in languages other than Italian or English.

### 2.3. Data Extraction

We first extracted the general characteristics of each eligible systematic review or meta-analysis, the first author’s name, the year of publication, the type of epidemiological design, the total size of the samples in all the studies included in a review. Then, we extracted the main findings of each study, including how exposure was measured and the overall results, and we summarized the meta-analytic estimates. Finally, we input details of the limitations of the study, and the authors’ recommendations. Two investigators (M.P., L.L.) independently searched the literature, assessed the eligibility of the papers they retrieved, and extracted the data. Disagreements were solved by discussion with a third investigator (A.B.).

### 2.4. Study Quality Assessment

The PRISMA (preferred reporting items for systematic reviews and meta-analyses) checklist, developed in 2009 [12,13], was used for a critical assessment of the internal validity of the reviews and meta-analyses considered. A checklist was compiled for each study included in the umbrella review (see Table S3).

## 3. Results

Our literature search identified 8564 results in Scopus and 2121 in PubMed (Figure 1). Then 2359 of the former studies and 618 of the latter were removed after applying publication and language filters. Another 6178 and 1503 studies were excluded after screening the abstracts and titles. In the end, 74 studies were included in the pre-selection phase, and 40 of them were deleted after reading the full text, leaving 34 studies. At this point, 14 further articles were drawn from the lists of references of the reviews considered. A total of 48 studies were tabulated for the purposes of our umbrella review: 32 meta-analyses, 4 pooled analyses, 5 systematic reviews, and 7 other reviews. The quality of these studies varied considerably: the meta-analyses scored a mean 20.1 points on the PRISMA checklist, the pooled analyses 15.5, the systematic reviews 15.2, and the other reviews 9 points.

Table 1 contains data concerning the general characteristics of the studies identified, Table 2 shows the exposure measures and main findings of the studies, Table 3 shows the conclusions and recommendations, and Tables S1 and S2 show summaries of the results of the studies.

### 3.1. Dietary Patterns

The umbrella review ultimately included five studies (two meta-analyses [14,15], one three-cohort analysis [16], two systematic reviews [17,18]) on the association between dietary patterns and BC risk. A diet was judged to be “healthy” if it included plenty of fiber and limited amounts of saturated fat, animal proteins, and refined sugars. The Mediterranean diet (MD) also contemplates a moderate consumption of red wine, and the use of extra-virgin olive oil for seasoning. These two (healthy and MD) eating patterns have been associated with a protective role against the development of BC [14,17,18] (Brennan, all studies combined: Odds Ratio (OR) 0.89, 95% CI 0.82–0.99). A systematic review [18], which included five cohort studies and three case-control studies, found that the case-control studies had identified an inverse association between MD and BC risk, in both pre- and post-menopausal women, but the cohort studies reported controversial results. One study that examined three cohorts using a standardized method found no association between a vegetable-based diet (characterized by high intakes of vegetables, legumes, fruit, pasta, fish, and oil) and BC risk in any of the cohorts [16]. This goes to show that the literature still does not offer a clear evidence of the association between a healthy diet and the risk of BC.

The Western diet includes large amounts of refined sugars, proteins, saturated fats, and alcohol, and—judging from case-control studies alone—it appears to be associated with a higher risk of BC (OR 1.31, 95% CI 1.05–1.63 [14]). On the other hand, when Brennan combined all types of study, there was no evidence of any difference in the risk of BC for women in the highest category of intake of Western/unhealthy dietary patterns compared with the lowest category (OR = 1.09, 95% CI: 0.98, 1.22).

### 3.2. Foods

Five studies (two meta-analyses [19,20], one systematic review [21], one pooled analysis [22] and one qualitative review [23]) examined the association between BC risk and red and processed meat consumption. Guo’s meta-analysis [19] found a positive association between the risk of BC and both red meat (summary RR 1.10, 95% CI 1.02–1.19) and processed meat (summary RR 1.08, 95% CI 1.01–1.15), and Taylor [20] reported an association between red meat consumption and a higher BC risk in premenopausal women (summary RR 1.24, 95% CI 1.08–1.42) [24]. On the other hand, the pooled study [22] found no significant associations between the intake of total meat, red meat or white meat, and BC risk.

It emerged from one meta-analysis [25] that a high weekly rate of egg consumption was associated with an increased risk of developing BC, that was only significant in women of postmenopausal age (RR estimates: 1.06; 95% CI 1.02–1.10).

Of the four studies that considered dairy food consumption (two meta-analyses [26,27], one pooled analysis [22], and one systematic review [28]), two reported an inverse association between the consumption of dairy products and the risk of BC [26,27].

Eleven of the studies analyzed the association between BC and the intake of soy foods, phytoestrogens, isoflavones, and lignans (six meta-analyses [9,29,30,31,32,33], three systematic reviews [22,28,34], and three qualitative reviews [35,36,37]). Three of these studies [29,32,34] demonstrated a significant inverse association between BC and a high lignans consumption in postmenopausal women, while two others [31,36] detected stronger evidence of this association in premenopausal women. The protective role of soy and phytoestrogens was recognized as significant in another seven studies [9,21,28,30,33,35,37].

Seven studies (three meta-analyses [24,33,38], two systematic reviews [21,28], one qualitative review [23], and one pooled analysis [39]) investigated dietary fiber: the three meta-analyses confirmed its protective effect against BC, the pooled analysis [39] found an inverse borderline association, while one of the systematic reviews [21] and the qualitative review [23] found no association between fruit fiber intake and the incidence of BC. The other systematic review only found a protective role for vegetable consumption, but no significant association with fruits or fruit fiber [28]. In another meta-analysis [40], Song reported a statistically significant association between citrus fruit consumption and the risk of developing BC (summary RR 0.90; 95% CI 0.85–0.96). A further meta-analysis [41] showed a significant inverse association between mushroom consumption and the risk of BC, in both premenopausal women (RR 0.96, 95% CI 0.91–1.00), and postmenopausal women (summary RR 0.94; 95% CI 0.91–0.97). The scientific evidence of any role for green tea in preventing BC is rather weak: one meta-analysis [42] and one systematic review [28] included in our umbrella review found no significant association, while a subgroup analysis showed a statistically significant inverse association in case-control studies (summary RR 0.56; 95% CI 0.38–0.83).

### 3.3. Nutrients

Eight of the studies (including four meta-analyses [33,43,44,45], two systematic reviews [21,28], one pooled analysis [46] and one qualitative review [23]) analyzed fat intake and the risk of BC. One meta-analysis [43] found no statistically significant association (summary RR estimate 1.11, 95% CI 0.91–1.36); one of the systematic reviews [29] and the qualitative review [23] obtained similar results. Another four studies found an increased risk of BC in women with a high intake of total fat [21,33,44] (Boyd et al. [44]) (summary RR 1.13, 95% CI 1.03–1.25) and saturated fat [46] (summary RR 1.19, 95% CI 1.00–1.19); one study found a positive association between a high intake of total fat and BC risk in postmenopausal women only [45] (summary RR 1.04, 95% CI 1.01–1.07).

Only one of the two meta-analyses included in our review [47,48] showed a modest increase in the risk of BC for women with dietary patterns associated with a high glycemic index (GI) or glycemic load (GL) [48]. Another two studies investigating the association between foods with a high GI or GL and the risk of BC also produced controversial results [21,28].

The consumption of polyunsaturated fatty acids (PUFA) was examined in seven studies (five meta-analyses [44,45,49,50,51], one systematic review [21], and one pooled analysis [46]). Two meta-analyses [49,50] found a significant protective association, three studies [44,46,51] reported a statistically insignificant inverse association, and another two studies [21,45] identified a higher risk of BC in women with a high PUFA consumption (summary RR: 1.07; 95% CI 1.01–1.14).

As for vitamin D and calcium, three studies (two meta-analyses [52,53] and one qualitative review [54]) considered these factors, and their protective effect was confirmed.

Three studies (two meta-analyses [55,56], and one qualitative review [57]) tested the association between folate consumption and the risk of BC, finding no significant inverse association overall. The only study that considered flavonoids and flavones (one meta-analysis [58]) concluded that the risk of BC was significantly reduced in women with a high overall consumption of flavonoids (RR 0.88; 95% CI 0.80–0.96), and flavones (RR 0.80; 95% CI 0.76–0.91). One meta-analysis [59] and one systematic review [28] investigated the relationship between carotenoid consumption and BC: the meta-analysis found that α-carotene and β-carotene consumption had a significant protective effect against BC, while the systematic review showed no such association. One systematic review [28] studied the association between BC and vitamins A, B, C and E, finding no such association in ten cohort studies; a positive association between vitamin E and BC emerged in one cohort study.

The overall results of the studies are summarized in Tables S1 and S2.

## 4. Discussion

Judging from this review, a higher intake of total meat, or red or processed meats, or of foods with a high GI, or eggs would seem to be associated with a higher risk of BC. Other foods, such as vegetables, soy and carotenoids, would seem to be inversely associated with BC risk. One meta-analysis demonstrated an inverse association between BC and the consumption of citrus fruits and mushrooms. Some nutrients also seem to be inversely associated with BC risk, including calcium, folate, vitamin D, lignans, and carotenoids. As concerns exposure to other dietary elements (polyunsaturated fatty acids, dairy foods), the evidence is still conflicting.

### 4.1. Dietary Patterns

Our findings indicate that most of the literature analyzed here attributed a protective role to the MD [19,51]. Research has shown that this type of diet is rich in antioxidants, which probably inhibit the synthesis and activity of growth factors promoting the development of cancer cells. A more recent meta-analysis of studies on postmenopausal BC [61] found an inverse association between MD and BC risk, when alcohol was excluded (summary Hazard Ratio (HR) 0.92; 95% CI 0.87, 0.98), while this association disappeared when alcohol was included. This might indicate that the MD could even be used as a primary BC prevention measure, especially in postmenopausal women. Some authors have said that calorie balance, adiposity control, and exercise are important to BC prevention too, as well as the composition and quality of the diet [62,63,64,65]. The MD also seems to have a beneficial influence against the risk of BC regardless of body weight and BMI [66,67,68,69]. The literature on the traditional MD has demonstrated that dietary fiber has multiple protective effects, which include inhibiting intestinal estrogen reabsorption, and modulating cholesterol levels and glucose release, thereby reducing BC risk [70]. The protective effect of fruit and vegetables seems to be linked to their high content of beneficial substances (vitamins, minerals, salicylates, phytosterols, glucosinolates, polyphenols, phytoestrogens, sulfides, lectins, etc.), which have an antioxidant action, preventing the activation of many carcinogens, suppressing spontaneous mutations, and protecting cellular structures and DNA against the oxidative damage generated by metabolic processes [70,71,72]. Leafy vegetables are rich in lutein, zeaxanthin, folates, vitamin A and carotenoids, which are antioxidant and also able to regulate estrogen metabolism and inhibit tumor growth [73,74]. Fruits seem to have an anti-carcinogenic potential due to their antioxidant properties; this is especially true of red fruits, which contain ellagic acid, quercitin and anthocyanins. These substances stimulate the mechanisms behind the elimination of toxic substances, inhibit angiogenesis, reduce inflammation, and promote cellular apoptosis mechanisms [71,75]. Some mushroom-derived substances, like polysaccharides, are known for their anti-tumor and immunomodulatory properties, for enhancing immune system activity and protecting against tumor recurrences [76]. A pooled analysis of eight large cohort studies did not strongly associate the intake of fruit and vegetables with the risk of BC, however [46]. Another comparative study of three cohorts also indicated that a diet rich in vegetables and fruits, but also characterized by other foods such as oil and fish, was not significantly related to a lower risk of BC [46].

The studies included in the present review indicated that the Western diet, which involves a high intake of refined sugars, saturated fats and alcohol, is strongly associated with an increased risk of BC [14,17]. This type of diet influences inflammatory processes, and induces an increase in adiposity and the production of growth factors and hormones (estrogen and testosterone).

Overall, however, the studies reviewed did not produce consistent results concerning specific dietary patterns and the risk of BC. Further studies could probably be conducted using machine learning techniques: this type of analysis could test clusters of foods not defined a priori—instead of prototypic dietary patterns—in terms of their association with BC risk, or BC prevention. In fact, a diet can include numerous foods that might have opposite effects, in which case analyzing preset eating patterns would be unable to produce evidence of any clear association.

### 4.2. Foods

The significant association demonstrated by the EPIC study [77] between red or processed meat consumption and a higher risk of BC, and BC-related mortality, was also confirmed in our umbrella review [19,20]. Apart from the related fat intake, various other factors appear to be involved in the carcinogenic potential of red and processed meat: the presence of carcinogens or their precursors (polycyclic aromatic hydrocarbons, heterocyclic aromatic amines, nitrosamines) produced by food treatments like cooking, salting or smoking; a high intake of animal proteins, with a potential increase in insulin-like growth factor (IGF) levels; hormone residues contained in meat from intensive farming; and the fact that intestinal fermentation of animal proteins raises the concentration of some polyamines, which are fundamental to cell proliferation.

A significant association also emerged between egg consumption and a higher risk of BC [24], but this relationship needs to be confirmed. The most plausible mechanism behind the carcinogenic potential of eggs might relate to their high cholesterol content (425 mg per 100 g) [24], given the recommended daily dose of 300 mg. Cholesterol is a precursor of steroid hormones, so it can influence estrogen activity and contribute to cellular inflammation, a crucial component in BC progression [78]. However, some evidence points to the association between eggs and BC being not necessarily due to cholesterol [79]: when fried at high temperatures, eggs also become a source of heterocyclic amines and several carcinogenic components, so their mutagenic activity can increase in some conditions (cooking at high temperatures and with inappropriate oil) [80].

Our umbrella review showed that studies consistently provided evidence of a protective effect of soy in the diet against the risk of BC [9,29,30,32,34,35,36], especially among postmenopausal women [28,31,33]. Phytoestrogens are structurally and functionally natural substances similar to estradiol, with a similar estrogenic activity. The evidence indicates a lower BC risk in consumers of soybean and phytoestrogen-containing plants, particularly in Asian populations (who are exposed to such foods from childhood), and in postmenopausal women. The protective effect of these foods is due to the agonist or antagonist actions against estrogens in breast tissue, reducing blood levels of estradiol [81,82] and consequently also the risk of estrogen receptor positive (ER+)/progesterone receptor positive (PR+) BC.

Finally, our umbrella review found that consuming lean dairy products was associated with a lower risk of BC [25,26], probably due to the protective effect of vitamin D and calcium [52,53,54]. Breast tissue has receptors to the biologically active form of vitamin D, calcitriol 1,25(OH)_2_D, which appears to be able to directly and indirectly control more than 200 genes, including those responsible for cell proliferation, malignant cell differentiation, apoptosis and angiogenesis [83]. In contrast, there are also reports of dairy product consumption leading to an increased estrogen hormone intake, which can enhance the penetrance of BC associated with Breast Related Cancer Antigens (BRCA) mutations [84]. In addition, milk has the potential to raise blood levels of growth factors, and a diet rich in animal protein is associated with high serum IGF-1 levels, which are strongly associated with a greater risk of BC [85,86].

The consumption of citrus fruits, rich in vitamin C, β-carotene, quercitin and folate, seems to have a very positive impact on health due to the antioxidant, immune-stimulant, and detoxifying properties, and a capacity to modulate insulin sensitivity and cholesterol levels [87]. In fact, our review found a 10% lower BC risk in women who consumed large amounts of citrus fruit.

### 4.3. Nutrients

A significant inverse association has been demonstrated between BC risk and consumption of carotenoids [88], which have antioxidant properties. These compounds, especially β-carotenes, are capable of binding and eliminating free radicals, and repairing DNA damage, inhibiting cell proliferation, inducing apoptosis, and suppressing angiogenesis [89]. Flavonoids have been acknowledged to have a role in protecting against and preventing non-communicable diseases [90,91,92,93], and neoplasms in particular, due to their potent antioxidant and DNA repairing activity. In this review, the dietary impact of various subclasses of flavonoids was analyzed, and it proved significant for flavonols, flavones, and flavan-3-ol swell classes, particularly after menopause. Folic acid, a soluble member of the vitamin B group, is needed for all DNA synthesis, repair and methylation reactions, so folate deficiency in the diet can negatively affect cell division and DNA repair mechanisms. The studies assessed in our umbrella review generally reported inconsistent results on the association between folic acid and BC, however.

Foods with a high GI, such as simple sugars, refined carbohydrates and starches, induce a rapid increase in blood glucose, and consequently stimulate insulin production. High levels of insulin cause the production of IGF-1 and testosterone, which are recognized as risk factors for BC. In addition, chronic hyperinsulinemia with associated insulin resistance has a key role in the etiology of BC as it induces the production of IGF-1, which is capable of causing mutagenic changes [94]. The studies included in our review [47,48] demonstrated no strong association between high GI food consumption and any increased risk of BC, however; this is probably due to reliability issues with the measurement indices adopted.

On the whole, our review identified a moderate risk of BC in women with a high total fat and/or saturated fat intake [32,44,46]. There was also evidence to support this higher BC risk applying especially to postmenopausal women [95,96,97], and the consumption of saturated fat is also associated with a higher likelihood of obesity [98,99,100,101,102,103,104]. Obesity and overweight, together with lack of exercise, are established risk factors for BC [105,106]. A diet rich in saturated fat also increases estrogen synthesis, leading to an increased cell proliferation and a consequently higher BC risk [107,108].

On the other hand, our review found that polyunsaturated fatty acids (PUFA) can have a role in reducing BC risk [49,50], although some studies found controversial results [21,45]. A possible explanation of the discrepancy of results concerns the fact that the two studies that found a protective effect of the PUFAs against the BC specifically examined the intake of n-3/n-6 PUFAs [49] and marine n-3 PUFA [50]. On the contrary, in the two studies [21,45] which gave an opposite result, the association between BC and the PUFA subtypes was not examined. The n-3 PUFA family compete for the same metabolic pathway with the n-6 PUFA family, which is associated with cell proliferation in breast tissue. The n-3 PUFA family reduces inflammation, controls triglyceride levels, and thus reduces the risk of BC via several mechanisms, i.e., by altering the composition of the phospholipid cell membranes, inhibiting arachidonic acid (ARA) metabolism and pro-inflammatory molecule production, and modulating the expression and function of various receptors, transcription factors, and signaling molecules [109]. Marine-derived n-3 PUFA would seem to have a protective effect against the development of BC [110], particularly in postmenopausal women, whereas n-6 PUFA contribute to carcinogenic mechanisms with the production of pro-inflammatory eicosanoids, such as prostaglandin E2, which is implicated in angiogenic processes and in the suppression of cancer cell apoptosis [109,111].

Overall, our study confirms the content of the third report from the World Cancer Research Fund [112], which found moderate evidence of the following: consuming non-starchy vegetables might reduce the risk of estrogen-receptor-negative (ER-) BC; consuming foods containing carotenoids, or adopting diets high in calcium might reduce the risk of BC in both premenopausal and postmenopausal women; and consuming dairy products might reduce the risk of BC, but only in premenopausal women. Our study also indicated that other foods and nutrients, including soy, folate, vitamin D, and lignans, seem to be inversely associated with BC.

This umbrella review of studies investigating the associations between diet, or specific foods or nutrients and BC reveals the weaknesses of the observational studies and reviews published on this topic (as shown in Table 3). The main shortcoming concerns measurement errors in dietary intake assessments, which bias the estimates of any such associations. Meta-analyses and systematic reviews of case-control studies may also be affected by recall and interview bias, often revealing associations that are not confirmed in cohort studies. Some studies also adjusted inconsistently for potential confounders (e.g., physical activity, often associated with both outcome and exposure), and this could result in residual confounding elements that would bias the estimates emerging from a meta-analysis.

## 5. Conclusions

Nutrition is one of the most modifiable aspects of lifestyle, and nutritional choices can affect people’s health and the risk of cancer. Active strategies are warranted, including educational/behavioral interventions in high-risk groups, to promote healthy eating habits.

## Figures and Tables

**Table 1:** Characteristics of studies included in the umbrella review.

Author_ Year	Continent or Region, n. of Studies	Search Range Applied	Total n. of Studies Included in the Review	Total Sample Size of all Studies Included in the Review	Type of Study	Study Design	Association/s Examined
**Characteristics of meta-analyses included in the umbrella review (32)**							
**Alexander 2010 [43]**	//	up to 2008	11 studies referring to 6 cohort studies	//	meta-analysis and review of epidemiological cohort studies	6 cohort studies	animal fat intake and breast cancer (BC)
**Aune 2012 [38]**	9 studies in America, 6 in Europe and 1 in Asia	from January 2006 to 31 August 2011	16 prospective studies (17 publications)	26,523 cases among 999,271 participants	systematic review and meta-analysis	16 cohort studies	high vs. low dietary fiber intake and BC risk
**Boyd 2003 [44]**	22 studies in Europe (including Russia), 15 in North America, 5 in Asia, 2 in Australia and 1 in South America (Uruguay)	from January 1966 to July 2003	45 studies	16,280 BC cases and 18,966 controls in case-control studies; 8735 BC cases out of 568,549 participants (total 25,015 BC cases and 580,000 controls)	meta-analysis of the published literature	31 case-control studies and 14 prospective studies (cohort and nested case-control studies)	dietary fat and BC risk
**Brennan 2010 [14]**	7 studies in North America, 5 in Europe, 2 in South America (Uruguay), 2 in Asia	up to November 2009	16 articles	13,885 BC cases out of 465,891 participants in cohort studies; 10,746 BC cases and 34,311 controls in case-control studies	systematic review and meta-analysis	8 case-control and 8 cohort studies	dietary patterns and BC risk
**Buck 2010 [29]**	16 studies in Europe, 6 in North America 1 in Asia and 1 in Australia	from 1997 to August 2009	24 articles, including 21 studies (all on lignan exposures—9 studies on calculated dietary plant lignans)	21,159 BC cases among 188,302 participants overall	meta-analysis	11 cohort studies and 10 case-control studies	lignans and BC risk
**Chen 2010 [52]**	//	up to July 2009	11 studies on vitamin D intake and 15 on calcium intake (26 studies in all)	//	meta-analysis	5 case-control and 6 cohort studies on vitamin D intake; 9 case-control and 6 cohort studies on calcium intake	vitamin D and calcium intake on BC risk
**Chen 2014 [55]**	20 studies in North America,10 in Europe, 9 in Asia, 1 in Australia, 1 in China 1 in Brazil	up to January 2014	50 eligible studies, 42 of which reported on folate intake and breast cancer	26,205 BC cases out of 744,068 participants in prospective studies; 16,826 BC cases and 21,820 controls in case-control studies	systematic review and meta-analysis	16 prospective studies (14 cohort and 2 nested case-control) on folate intake; 26 case-control studies on folate intake (8 studies on blood folate levels)	folate intake and overall BC risk
**Dong 2011 (a) [29]**	8 studies in Europe, 4 studies in Asia, 2 studies in USA	up to September 2010	14 studies on BC incidence	369,934 participants and 5828 cases of BC	meta-analysis of prospective studies (nested case-control and cohort studies)	6 nested case control studies and 8 cohort studies	soy isoflavone consumption and BC risk
**Dong 2011 (b) [25]**	9 studies in USA, 8 in Europe, 1 in Japan	up to January 2011	18 studies	24,187 BC cases and 1,063,471 participants	meta-analysis of prospective studies (nested case-control and cohort studies)	18 cohort studies; 12 studies on total dairy food intake and 13 on milk intake	dairy product consumption and BC risk
**Gandini 2000 [38]**	14 studies in Europe, 8 in North America, 3 in Asia, 1 in Australia	1982–1999	26 studies focusing on vegetables (17), fruits (13), beta-carotene (11) and vitamin C (9)	23,038 cases in all	meta-analysis	5 cohort studies and 21 case-control studies	consumption of fruit and vegetables, and associated micronutrients, and risk of BC
**Gissel 2008 [53]**	//	up to June 2007	6 studies	78,712 women	meta-analysis	epidemiological studies (cross-sectional, case-control, or cohort) and randomized controlled trials (RCTs)	vitamin D intake and BC
**Guo 2015 [19]**	6 studies in the USA, 6 in Europe, 1 in North America and Western Europe, and 1 in Asia	up to October 2014	14 prospective studies	1,588,890 participants and 31,552 BC cases	meta-analysis	14 cohort/nested case-control studies	red meat and processed meat consumption and BC risk
**Hu 2012 [59]**	//	January 1982–May 2011	33 studies	//	meta-analysis and meta-regression	24 case-control studies, 1 nested case-control study, 2 case-cohort studies and 6 cohort studies	carotenoids and BC risk
**Hui 2013 [58]**	5 studies in Europe, 5 in North America (4 in the USA) and 2 in Asia (China)	up to July 2012	12 studies included	9513 cases and 181,906 controls	meta-analysis of epidemiological studies	6 cohort studies, 6 case-control studies	flavonoids, flavonoid subclasses and BC risk
**Larsson 2007 [56]**	12 studies in North America (10 in the USA), 7 in Europe, 2 in Asia, 1 in South America (Uruguay) and 1 in Australia	from January 1966 to November 2006	23 studies (21 assessing dietary folate intake)	8367 BC cases out of 302,959 participants in cohort studies; 8558 BC cases and 10,812 control subjects in case-control studies	meta-analysis	9 cohort studies and 14 case-control studies	dietary folate intake and BC risk
**Li 2014 [41]**	8 case-control studies in Asia, 2 cohort studies in Europe	up to 2013	7 articles including 10 studies	2313 BC cases and 2387 controls in case-control studies; 4577 cases among 1,748,623 follow-up person/years	meta-analysis	2 cohort and 8 case-control studies	dietary mushroom intake and BC risk
**Mulholland 2008 [47]**	8 studies in North-Central America, 5 in Europe and 1 Australia	up to May 2008	14 studies	17,673 BC cases out of 492,011 participants	systematic review and meta-analysis	10 cohort, 4 case-control studies	dietary glycemic index, glycemic load and BC risk
**Mullie 2016 [48]**	6 studies in North America, 4 in Europe, 1 in Australia and 1 in China	up to December 2011	12 studies	20,973 BC cases out of 773,971 subjects	meta-analysis of prospective cohort studies	12 cohort studies	high glycemic index, glycemic load and BC risk
**Schwingshackl 2014 [15]**	7 studies in Europe, 4 in North America	up to January 2014	11 studies on BC risk	489,109 participants in cohort studies; 4990 BC cases and 5446 controls in case-control studies	systematic review and meta-analysis	5 case-control and 6 cohort studies	Mediterranean diet and BC risk
**Seely 2005 [42]**	4 studies in Asian populations (Japanese, Chinese or Filipino) in Asia, 1 in Asian populations of the USA	from 1966 to November 2004	7 studies (5 prevention studies examining risk of primary BC and green tea consumption)	857 primary BC cases and 1519 controls in case-control studies; 115,601 participants in cohort studies	systematic review and meta-analysis	3 cohort studies and 2 case-control studies	green tea consumption and BC risk
**Si 2014 [25]**	5 studies in Asia, 4 in Europe, 2 in the USA, 1 in South America and 1 in both Europe and America	up to February 2013	13 studies	19,280 BC cases among 811,555 participants in cohort studies; 13,949 cases and 37,883 controls in case-control studies	meta-analysis	5 cohort studies and 8 case-control studies	egg consumption and BC risk
**Song 2013 [40]**	3 studies in China, 2 in the USA, 1 in Australia	up to January 2012	6 studies	8393 participants: 3789 cases and 4705 controls	quantitative systematic review and meta-analysis	6 case-control studies	association between citrus fruit intake and BC risk
**Taylor 2009 [20]**	5 studies in North America, 2 in Europe, 2 in Asia and 1 in South America	from 1966 to 2009	10 studies	1842 BC cases and 2124 controls in case-control studies; 2024 BC cases among 192,627 participants in cohort studies	meta-analysis	6 case-control studies, 1 nested case-control and 3 cohort studies	red meat intake and BC risk
**Trock 2006 [31]**	8 studies in Asian populations and 10 in Western populations	from 1978 to 2004	18 studies	9182 BC cases	meta-analysis	12 case-control and 6 cohort or nested case-control studies	soy intake and BC risk
**Turner 2011 [45]**	25 studies in North America (USA), 18 in Europe, 2 in South America (Uruguay), 6 in Asia, and 1 in Australia	up to May 2010	52 studies	44,070 BC cases among 1,659,913 participants/controls	meta-analysis	25 cohort studies and 27 case-control studies	fat intake and BC risk
**Velentzis 2009 [32]**	6 studies conducted in North America, 6 in Europe	up to September 2008	23 studies, 12 of them on plant lignan intake	12,430 BC cases among 258,984 participants	meta-analyses	5 cohort and 7 case-control studies	lignans and BC risk
**Wu 2008 [9]**	8 studies in Asia and Asian Americans, 11 in Western populations	//	19 studies	//	meta-analysis	14 case-control studies and 5 cohort/nested case-control studies	soy exposure and BC risk
**Wu 2015 [33]**	All studies on Chinese populations	up to June 2013	22 studies	23,201 subjects: 10,566 in the experimental group (cases) and 12,635 controls	systematic review and meta-analysis	21 case-control studies and 1 cohort study	dietary factors and BC risk
**Yang 2014 [49]**	4 studies in the USA, 5 in Europe and 2 in Asia	up to April 2013	11 prospective studies	8331 BC events among 274,135 adult women	meta-analysis	6 nested case-control studies and 5 cohort studies	n-3/n-6 PUFAs and risk of BC
**Zang 2015 [27]**	11 cohort studies in the USA, 10 in Europe, 1 in Japan; 5 case-control studies in Asia	up to January 2014	27 studies	37,925 BC cases among 1,566,940 participants in cohort studies and 7418 incident BC cases among 33,372 participants in case-control studies	systematic review and meta-analysis	22 cohort studies and 5 case-control studies	dairy intake and BC
**Zheng 2013 [50]**	11 studies in USA, 11 in Europe and 4 in Asia	up to December 2012	26 publications on 21 prospective studies: (14 on fish intake and 19 on marine n-3 PUFA)	20,905 BC cases and 883,585 participants	meta-analysis	26 cohort studies (8 nested case-control and 2 case-cohort studies)	intake of fish and marine omega-3 polyunsaturated fatty acids (n-3 PUFA)and risk of BC
**Zhou 2015 [51]**	6 studies in Europe, 5 in the USA and 1 in Asia (China)	up to November 2014	12 studies, 2 of which reported two separate outcomes (pre- and post-menopausal), for a total of 14 outcomes included in the meta-analysis	10,410 BC events among 358,955 adult females	meta-analysis	8 cohort studies and 4 nested case-control studies	linoleic acid and BC risk
**Characteristics of pooled analyses included in the umbrella review (4)**							
**Mannisto 2005 [16]**		//	3 cohort studies	77,037 participants and 3271 BC cases	pooled analysis	3 cohort studies in the DIETSCAN project: Netherlands Cohort Study on diet and cancer (NCLS), the Italian “Ormoni e Dieta nella Eziologia dei Tumori”(ORDET) and the Swedish Mammography Cohort (SMC)	dietary pattern and BC risk
**Missmer 2002 [22]**	North America and Western Europe	//	8 studies	7379 BC cases out of 351,041 women	pooled analysis	8 cohort studies	meat and dairy food consumption and risk of BC
**Smith-Warner 2001 (a) [46]**	7 studies in North America, 2 in Europe	up to 2001	8 studies (9 in all, because the Nurse’s Health Study was divided in two)	7329 BC cases out of 351,821 participants	pooled analysis of cohort studies	pooling project: 9 prospective studies (8 analyzed as nested case-control studies, 1 used a case-cohort design)	types of dietary fat and BC risk
**Smith-Warner 2001 (b) [39]**	7 studies in North America, 2 in Europe	up to 2001	8 studies (9 in all, because the Nurse’s Health Study was divided in two)	7377 incident BC cases among 351,825 participants	pooled analysis of cohort studies	pooling project: 9 prospective studies (8 analyzed as nested case-control studies, 1 used a case-cohort design)	intake of fruits and vegetables and BC risk
**Characteristics of systematic reviews included in the umbrella review (5)**							
**Albuquerque 2013 [17]**	8 studies in Europe, 9 in North America, 5 in Asia, 4 in South America, 1 in Africa, and 1 in Oceania	up December 2012	26 articles	584,437 women and 28,962 incident cases of BC	systematic review	11 cohort studies and 15 case-control studies	dietary patterns and BC risk
**Farsinejad-Marj 2015 [18]**	7 studies in Europe, 1 in America	up to April 2015	8 studies	502,253 participants	systematic review	5 cohort studies; 3 case-control studies	Mediterranean diet and BC risk
**Michels 2007 [28]**	not clearly defined	January 1950–May 2005	21 studies on fat intake, 8 on fruit and vegetables, 11 on carbohydrates, 11 on antioxidants, 12 on dairy products, 2 on vitamin D, 5 on soy, 6 on green tea, 1 on heterocyclic amines, 2 on adolescent diet	not clearly defined	systematic review	73 cohort studies, 3 meta-analyses, 3 pooled analyses	diet and BC
**Mourouti 2013 [34]**	//	from January 2000 to April 2013	29 studies, 12 on populations with a high consumption of soy	//	systematic review	10 cohort, 3 nested case-control and 16 case-control studies	soy food consumption and BC risk
**Mourouti 2015 [21]**	//	from January 2002 to August 2012	95 studies on: fruit and vegetable (11), meat (14), soy (24), dietary fiber (9), dietary carbohydrates (16), dietary lipids (21)	fruit and veg: 16,110 cases among 122,933 participants meat: 31,918 cases among 813.021 participants soy: 9064 cases among 324,345 participants dietary fiber: 15,880 cases among 641,322 participants fat: 24,788 cases among 951,479 participants diet carbohydrates (studies showing a positive association): 4332 cases among 146,692 participants	systematic review	42 case-control studies, 51 cohort studies and 2 dietary intervention RCTs	diet and BC
**Characteristics of not systematic reviews included in the umbrella review (7)**							
**Cui 2006 [54]**	vitamin D: 7 studies in North America (6 in USA), 1 in Europe (Switzerland) Calcium: 9 studies in Europe, 2 in North America, and 1 in Asia (China)	not defined	20 studies (8 on dietary vitamin D and 12 on calcium intake)	vitamin D: 7947 BC cases out of 218,776 subjects; calcium: 11,378 BC cases out of 16,764 subjects	not-systematic review	vitamin D: 5 prospective studies (4 cohort and 1 nested case-control) and 3 case control studies; calcium: 3 cohort and 9 case-control studies	association between vitamin D and calcium and BC risk
**Duffy 2007 [35]**	5 studies in Europe, 8 in Asia, 6 North America	//	21 studies	12,472 BC cases and 35,513 controls in case-control studies; 1799 BC cases among 206,030 participants in cohort studies	not-systematic review	6 cohort studies and 15 case-control studies	phytoestrogen intake and BC risk
**Eichholzer 2001 [57]**	6 studies in North America, 1 in South America, and 1 in Europe	//	8 studies	9420 BC cases	not-systematic review	5 case control studies and 3 nested case-control studies	folate intake and BC risk
**Hanf 2005 [23]**	//	//	13 prospective studies on fat intake; 10 prospective studies on meat consumption	//	not-systematic review	cohort studies and interventional trials	foods, nutrients and BC risk
**Lof 2006 [36]**	5 studies in North America, 3 in Europe (1 of them on an Asian population)	from 1966 to September 2006	7 studies	4741 BC cases among 134,100 participants	not-systematic review	5 case-control studies and 2 cohort studies	dietary phytoestrogens intake and BC risk
**Peeters 2003 [37]**	2 studies in North America and 11 on Asian populations	up to November 2011	13 studies on dietary phytoestrogens	5954 BC cases out of 27,162 participants	not-systematic review	9 case-control studies and 4 cohort studies	phytoestrogens and BC risk
**Rossi 2014 [60]**	//	1975–2013	175 pertinent articles: 18 for proteins, 18 for carbohydrates, 18 for dietary fat, 33 for polyphenols and phytoestrogen, 18 for fruit and vegetables, 11 for lycopene, 44 for vitamins and oligo elements, 8 for alcohol	//	not-systematic review	//	impact of dietary factors in BC risk

**Table 2:** Results of studies included in the umbrella review.

**Author_ Year**	**Menopausal Status**	**Exposure Measure**	**Overall Results of Review**	**Statistical Method**	**Summary Estimates and Related 95% CI**	***p*-value**
**Results of meta-analysis included in the umbrella review (32)**						
**Alexander 2010 [43]**	pre- and post-menopause	animal fat intake (highest vs. lowest categories)	no statistically significant association was found between animal fat intake (comparing highest vs. lowest category) and BC risk.	random effect model	summary relative risk (RR) estimates: 1.11 (0.91–1.36)	//
**Aune 2012 [38]**	pre- and post-menopause	dietary fiber, fruit fiber, vegetable fiber, cereal fiber, soluble and insoluble fiber	an inverse association was found between dietary fiber intake and BC risk; the association appeared to be most pronounced in studies with high levels (>25 vs. <25 g/day) or large ranges (>13 vs. <13 g/day) of fiber intake.	random effects model was used to calculate summary RRs and 95% CIs	summary RR (high vs. low intake):	//
					- dietary fiber 0.93 (0.89–0.98)	
					- fruit fiber 0.95 (0.86–1.06)	
					- vegetable fiber 0.99 (0.92–1.07)	
					- cereal fiber 0.96 (0.90–1.02)	
					- soluble and insoluble fiber 0.91 (0.84–0.99)	
**Boyd 2003 [44]**	//	dietary fat: total fat, saturated fat and monounsaturated fat; foods containing animal fat: meat, milk and cheese	a higher fat intake is associated with a higher risk of BC, with a significant positive association confirmed for total fat and saturated fat; meat and cheese consumption was also associated with a statistically significant increase in BC risk.	random effects model	summary RR (highest vs. lowest categories of intake) for all studies combined	//
					- total fat 1.13 (1.03–1.25)	
					- saturated fat 1.19 (1.06–1.35)	
					- monounsaturated fat 1.11 (0.96–1.28)	
					-polyunsaturated fat 0.94 (0.80–1.10)	
					- meat consumption 1.17 (1.06–1.29)	
					- milk consumption 1.12 (0.88–1.43)	
					- cheese consumption 1.26 (0.96–1.66)	
**Brennan 2010 [14]**	//	dietary patterns (DP): “prudent/healthy” vs. “Western/unhealthy”	no overall association was found for Western/unhealthy DP and BC risk; results of combined case-control studies showed that Western DP was positively associated with BC risk (highest vs. lowest category of intake); there was evidence of a lower risk of BC (highest vs. lowest category of intake) associated with a prudent/healthy DP in all studies and in pooled cohort studies.	random effects model	summary RR estimates (highest vs. lowest categories of intake):	
					Western/unhealthy diet overall 1.09 (0.98–1.22);	0.11
					- case-control combined 1.31 (1.04–1.63);	0.02
					- cohort combined 0.99 (0.90–1.08)	0.82
					Prudent/healthy diet overall 0.89 (0.82–0.99);	0.02
					- case-control combined 0.84 (0.67–1.04);	0.12
					- cohort combined 0.93 (0.88–0.98)	0.01
**Buck 2010 [29]**	pre- and post-menopause	lignan exposure, plant lignan intake	lignan exposure was not associated with a significantly lower risk of BC; but a high lignan intake in postmenopausal women was associated with a significant reduction in risk of BC.	random effects model and fixed effects model	pooled estimate (highest vs. lowest quantile)	//
					overall 0.92 (0.81–1.02)	
					- premenopause 0.87 (0.66–1.08)	
					- postmenopause 0.86 (0.78–0.94)	
					dietary plant lignans 0.94 (0.82–1.05)	
					- premenopause 1.01 (0.87–1.15)	
					- postmenopause 0.86 (0.77–0.94)	
**Chen 2010 [52]**	pre- and post-menopause	dietary calcium and vitamin D intake	a statistically significant overall inverse association was found between dietary vitamin D intake and BC risk; high vitamin D intake was associated with a 17% decrease in BC risk in premenopausal women; a statistically significant overall inverse association was found between dietary calcium intake and BC risk.	a random effects model if there was significant heterogeneity among studies; if not, a fixed effects model was acceptable	summary RRs (highest vs. lowest category):	//
					- vitamin D intake overall 0.91 (0.83–1.00)	
					premenopause 0.83 (0.73–0.95)	
					postmenopause 0.94 (0.83–1.07)	
					- calcium intake overall 0.81 (0.72–0.90)	
					premenopause 0.72 (0.55–0.95)	
					postmenopause 0.95 (0.79–1.14)	
**Chen 2014 [55]**	pre- and post-menopause	total folate intake, dietary folate intake and folate supplement intake	no significant association between dietary folate intake and BC risk in meta-analysis of prospective studies, but a significantly negative correlation emerged from the case control studies, particularly for postmenopausal women; more dietary folate may reduce the BC risk in populations of Europe, Australia or Asia (not in the USA).	inverse variance weighting method; random effects model	summary RRs (highest vs. lowest category) in prospective studies:	//
					total folate 0.97 (0.87–1.08);	
					dietary folate (DF) 0.95 (0.87–1.03)	
					- DF premenopause 1.02 (0.62–1.67)	
					- DF postmenopause 0.94 (0.81–1.08)	
					OR (highest vs. lowest category) in case-control studies:	
					total folate 0.87 (0.61–1.23)	
					dietary folate (DF) **0.79** (0.67–0.92)	
					DF premenopause 0.78 (0.53–1.14)	
					DF postmenopause **0.73** (0.59–0.92)	
**Dong 2011 (a) [30]**	pre- and post-menopause	dietary soy isoflavone intake	soy isoflavone intake was associated with a significantly reduced risk of BC in Asian populations, but not in Western populations; a significant inverse association between soy isoflavone intake and risk of BC emerged in postmenopausal but not in premenopausal women.	random or fixed effects model	summary RR:	//
					all women 0.89 (0.79–0.99)	
					Asian countries 0.76 (0.65–0.86)	
					Western countries 0.97 (0.87–1.06)	
					premenopause 0.90 (0.64–1.15)	
					postmenopause 0.78 (0.63–0.93)	
**Dong 2011 (b) [26]**	pre- and post-menopause	total dairy food defined as skimmed/low-fat milk, whole/high-fat milk, yogurt, cottage cheese, butter, and other dairy products	for total dairy food consumption, a significantly inverse association with BC risk was confirmed, and was stronger for low-fat dairy food and for premenopausal women; for milk consumption only low-fat milk was significantly associated with a lower BC risk; no association in postmenopausal women.	random effects model	summary RR (highest vs. lowest intake)	//
					total dairy food 0.85 (0.76–0.95)	
					milk 0.90 (0.80–1.02)	
					total dairy food	
					- premenopause 0.79 (0.63)	
					- postmenopause 0.92 (0.83–1.01)	
					total milk	
					- premenopause 0.79 (0.60–1.02)	
					- postmenopause 1.01 (0.94–1.09)	
**Gandini 2000 [38]**	//	vegetable and fruit consumption, and 2 related micronutrients: vitamin C and beta-carotene	high vegetable consumption, and high vitamin C and beta-carotene intake had a significant protective effect against BC; pooled estimates for fruit intake indicated that this food group may have a protective effect, but without statistical significance.	random effects model fixed effects model	summary RR (high vs. low consumption)	//
					- vegetables 0.75 (0.66–0.85)	
					- fruits 0.94 (0.79–1.11)	
					- vitamin C 0.80 (0.68–0.95)	
					- beta-carotene 0.82 (0.76–0.91)	
**Gissel 2008 [53]**	pre- and post-menopause	vitamin D consumption	no association between vitamin D levels and BC risk.	random effects model	summary RR: 0.98 (0.93–1.03) analysis restricted to intake <400 International Units (IU)/day vs. lower intake:	//
					RR 0.92 (0.87–0.97)	
**Guo 2015 [19]**	pre- and post-menopause	red and processed meat consumption	higher red and processed meat intake was associated with higher risk of BC.	random effects model	summary RR of BC (highest vs. lowest consumer categories) were: 1.10 (95% confidence interval (CI), 1.02–1.19) for red meat, and 1.08 (95% CI, 1.01–1.15) for processed meat intake; estimated summary RR were 1.11 (1.05–1.16) for an increase of 120 g/day of red meat, and 1.09 (1.03–1.16) for an increase of 50 g/day of processed meat	//
**Hu 2012 [59]**	pre- and post-menopause	dietary α-carotene; dietary β-carotene; β-cryptoxanthin; lutein+ zeaxanthin; lycopene	a significant inverse association between α-carotene consumption and BC risk, in both cohort and case-control studies; a trend towards a protective effect of β-carotene; no significant association between β-cryptoxanthin, lutein+zeaxanthin and BC risk; lycopene may have a protective effect against BC (significant inverse association in case-control studies).	random effects model	summary RR among cohort studies:	
					α-carotene 0.91 (0.85–0.98)	0.01
					β-carotene 0.94 (0.88–1.00)	0.05
					β-cryptoxanthin 1.03 (0.96–1.11)	0.39
					lutein+zeaxanthin 0.94 (0.87–1.02)	0.13
					lycopene 0.99 (0.93–1.06)	0.77
					summary OR among case-control studies:	
					α-carotene 0.82 (0.70–0.97)	0.02
					β-carotene 0.75 (0.67–0.85)	<0.01
					β-cryptoxanthin 0.95 (0.80–1.13)	0.54
					lutein+zeaxanthin 0.79 (0.66–0.94)	0.01
					lycopene 0.71 (0.56–0.92)	0.01
**Hui 2013 [58]**	pre- and post-menopause	flavonoid subclasses: flavonols (onions, broccoli, tea, fruit); flavones (herbs, parsley, chamomile); flavonones (citrus fruits, oranges, grapefruit); flavan-3-ols (dark chocolate, apples, grapes, red wine, green tea); anthocyanidins (berries, cranberries, black currants, blueberries); isoflavones (soy and soy products)	BC risk significantly lower in women with high intake of flavonols and flavones; no significant association with flavan-3-ols, flavonones, anthocyanins or total flavonoids. Summary RRs of studies stratified by menopausal status suggest that flavonols, flavones and flavan-3-ols are associated with a significantly lower risk of BC in postmenopausal, but not in premenopausal women.	random and fixed effects model	summary RR (highest vs. lowest consumption):	//
					flavonols 0.88 (0.80–0.96)	
					- postmenopause 0.92 (0.85–0.99)	
					flavones 0.83 (0.76–0.91)	
					- postmenopause 0.86 (0.77–0.94)	
					flavan-3-ols 0.93 (0.84–1.02)	
					- postmenopause 0.90 (0.83–0.98)	
					flavonones 0.95 (0.88–1.03)	
					anthocyanidins 0.97 (0.87–1.08)	
**Larsson 2007 [56]**	pre- and post-menopause	dietary folate intake	no significant association between dietary folate intake and BC risk in prospective studies; in case-control studies, high vs. low dietary folate intake was associated with a significantly different risk of BC (lower in the former).	random effects model	summary estimates (highest vs. lowest dietary folate intake categories):	//
					cohort studies: RR 0.96 (0.87–1.05)	
					case-control studies: Odds Ratio (OR) 0.73 (0.64–0.83)	
**Li 2014 [41]**	pre- and post-menopause	dietary mushroom intake	a significant inverse association between mushroom consumption and BC risk	random and fixed effects model	summary RR (highest vs. lowest dietary mushroom intake categories):	//
					premenopause 0.96 (0.91–1.00)	
					postmenopause 0.94 (0.91–0.97)	
					overall: 0.97 (0.96–0.99)	
**Mulholland 2008 [47]**	pre- and post-menopause	glycemic index (GI) and glycemic load (GL)	no strong associations between high vs. low GI and BC risk; a positive association emerged once analysis was restricted to more robust measures of dietary intake, for both premenopausal and postmenopausal women.	//	summary RR in cohort studies (high vs. low GI)	//
					premenopause RR 1.20 (1.01–1.43)	
					postmenopause RR 1.10 (1.02–1.19)	
**Mullie 2016 [48]**	pre- and post-menopause	dietary pattern with high GI or GL	women with high GI or GL had a small, 5–6% increase in BC risk.	random effects model	summary RR (high vs. low) for GI 1.05 (1.00–1.11)	//
					- premenopause 1.04 (0.86–1.27)	
					- postmenopause 1.05 (0.98–1.13)	
					for GL 1.06 (1.00–1.13)	
					- premenopause 1.23 (0.75–2.00)	
					- postmenopause 1.05 (0.97–1.13)	
**Schwingshackl 2014 [15]**	//	Mediterranean diet (MD; fruits, vegetables, legumes, olive oil, nuts, plant protein and whole grains, fish; moderate red wine consumption and low amounts of red meat, poultry and dairy products)	case-control studies showed a significant association between greater adherence to MD and lower BC risk; cohort studies showed no significant association between BC risk and MD. Overall, MD was not associated with a significant reduction in BC risk.	random effects model	pooled RR (highest vs. lowest adherence to MD category):	
					total 0.95 (0.84–1.06)	0.35
					cohort 1.01 (0.88–1.16)	0.89
					case-control 0.82 (0.69–0.97)	0.02
**Seely 2005 [42]**	//	green tea (dried leaves of Camellia sinensis with minimal oxidation of herbs, polyphenols and catechins)	random effects model revealed no significant association between green tea consumption and BC risk; fixed effects model in case-control studies showed a significant inverse association.	random effects model fixed effects model	pooled RR (highest vs. lowest consumption)	
					cohort studies 0.89 (0.71–1.10)	0.28
					case-control studies 0.44 (0.14–1.31)	0.14
					case-control studies 0.57 (0.38–0.86)	0.007
**Si 2014 [25]**	pre- and post-menopause	egg consumption quartiles: 1 a week (reference) ≥1, <2 a week ≥2, ≤5 a week > 5 a week	higher egg consumption was associated with higher BC risk; subgroup analyses showed this association in cohort studies, among Europeans, Asian populations, postmenopausal women, and consumers of ≥2 and ≤5 eggs/week.	random effects model	pooled adjusted RR	
					overall 1.04 (1.01–1.08)	0.02
					case control 1.06 (0.97–1.15)	0.21
					cohort 1.04 (1.00–1.08)	0.05
					postmenopause 1.06 (1.02–1.10)	0.01
					premenopause 1.04 (0.98–1.11)	0.2
					≥2 and ≤5/week (ref. <1/week)	
					RR 1.10 (1.02–1.17)	0.01
**Song 2013 [40]**	not stated	citrus fruit intake, yellow and orange fruit, oranges or tangerines	a higher intake of citrus fruit may reduce BC risk.	fixed effects model	summary OR (highest vs. lowest intake group): 0.90 (0.85–0.96)	//
**Taylor 2009 [20]**	premenopause	red meat consumption	a significant positive association was found overall between high of red meat intake and BC risk among premenopausal women; case-control studies confirmed a strong association between red meat and BC risk, while cohort studies found no statistically significant association between them.	//	summary RR (highest vs. lowest red meat intake):	//
					overall 1.24 (1.08–1.42)	
					cohort studies 1.11 (0.94–1.31)	
					case-control studies 1.57 (1.23–1.99)	
**Trock 2006 [31]**	pre- and post-menopause	soy protein (grams of soy protein consumed daily)	among all women, high soy intake was modestly associated with a lower BC risk; the association was not significant for Asian women; among 10 studies stratified by menopausal status, the inverse association between soy exposure and BC risk was stronger premenopause than postmenopause.	random effects model and fixed effects model (in premenopausal women)	pooled OR (high vs. low soy intake)	//
					all women 0.86 (0.75–0.99)	
					premenopause 0.70 (0.58–0.85)	
					postmenopause 0.77 (0.60–0.98)	
**Turner 2011 [45]**	premenopause (7), postmenopause (20), and both (25)	total fat, saturated fat (SAT), monounsaturated fat (MUFA), polyunsaturated fat (PUFA)	cohort studies found significant summary RR between PUFA and BC; case-control studies found no association between fat and BC; postmenopausal women had a significant association between total fat, PUFA intake and BC; an inverse association between total intake of all fats and BC was seen in premenopausal women, but was not significant.	random effects model	pooled RR (highest vs. lowest quartile of consumption)	
					total fat 1.01 (0.99–1.03)	non-significant (NS)
					SAT 1.00 (0.95–1.05)	NS
					MUFA 0.99 (0.95–1.05)	NS
					PUFA 1.06 (1.01–1.14)	0.03
					premenopause 0.97 (0.94–1.01)	NS
					postmenopause 1.04 (1.01–1.07)	0.004
**Velentzis 2009 [32]**	pre- and post-menopause	plant lignan intake	no significant association between plant lignan intake and BC risk, despite a slight protective effect; when menopausal status was considered, a statistically significant risk reduction was seen for the highest plant lignan intake in postmenopausal women.	random effects model	combined OR (highest vs. lowest plant lignan intake)	
					overall 0.93 (0.83–1.03)	0.15
					postmenopause 0.85 (0.78–0.93)	< 0.001
**Wu 2008 [9]**	pre- and post-menopause	soy intake - isoflavone Asian (highest >20 mg/day - lowest <5 mg/day) Western (highest >0.8 mg/day - lowest <0.15 mg/day)	a statistically significant 29% reduction in BC risk associated with high soy intake in Asian populations (high consumers); both premenopausal and postmenopausal Asian women consuming large amounts of soy had a lower BC risk; no significant association was found in Western countries.	random effects model	pooled OR (highest vs. lowest soy intake)	//
					Asian 0.71 (0.60–0.85)	
					- premenopause 0.65 (0.50–0.85)	
					- postmenopause 0.63 (0.46–0.85)	
					Western 1.04 (0.97–1.11)	
**Wu 2015 [33]**	//	dietary factors: vegetables, fruits, soy food, or dietary fat	high consumption of fruits, vegetables and soy foods was significantly inversely associated with a lower BC risk in Chinese women; higher fatty food consumption appeared to be related to higher risk of BC in Chinese women.	random effects model	combined OR (high vs. low intake)	
					vegetables 0.77 (0.62–0.96)	0.02
					fruit 0.68 (0.49–0.93)	0.02
					soy food 0.68 (0.50–0.93)	0.02
					fat 1.15 (1.01–1.30)	0.03
**Yang 2014 [49]**	pre- and post-menopause	dietary polyunsaturated fatty acid intake (n-3/n-6 PUFA)	intake ratio of n-3/n-6 PUFA was inversely associated with BC risk for the highest vs. lowest quantiles of the study population.	random effects model	pooled RR (highest quantile vs. lowest)	//
					overall diet 0.90 (0.82–0.99)	
					premenopause 0.80 (0.43–1.48)	
					postmenopause 0.85 (0.75–0.97)	
**Zang 2015 [27]**	pre- and post-menopause	dairy food consumption: high (>600 g/day) modest (400–600 g/day) low (<400 g/day) dairy food items (milk, cheese, butter) converted into grams of protein per day	higher dairy food consumption was associated with a statistically significant lower risk of BC, in both cohort and case-control studies; there was a marginally lower BC risk in premenopausal women.	random effects model	combined estimates (highest dairy consumption vs. lowest)	//
					cohort RR 0.90 (0.83–0.98)	
					- premenopause 0.88 (0.77–1.00)	
					- postmenopause 0.94 (0.86–1.02)	
					case-control OR 0.74 (0.62–0.88)	
**Zheng 2013 [50]**	pre- and post-menopause	dietary intake of marine n-3 polyunsaturated fatty acids (PUFA)	greater marine n-3 PUFA intake was associated with a 14% reduction in BC risk; no significant association for fish intake or exposure to alpha-linoleic acid.	random effects model	fish intake (highest vs. lowest category)	//
					overall 1.03 (0.93–1.14)	
					- premenopause 1.04 (0.91–1.20)	
					- postmenopause 1.08 (0.92–1.27)	
					marine n-3 PUFA (highest vs. lowest)	
					overall 0.86 (0.78–0.94)	
					- premenopause 0.96 (0.78–1.18)	
					- postmenopause 0.88 (0.76–1.00)	
**Zhou 2015 [51]**	pre- and post-menopause	linoleic acid	both dietary linoleic acid and overall linoleic acid level were associated with a lower risk of BC, but none of the associations were statistically significant.	random and fixed effects model	exposure assessment (highest vs. lowest category of linoleic acid)	//
					overall 0.98 (0.93–1.04)	
					dietary linoleic acid 0.96 (0.92–1.01)	
					premenopause 0.64 (0.20–2.09)	
					postmenopause 1.01 (0.94–1.08)	
**Results of pooled analyses included in the umbrella review (4)**						
**Mannisto 2005 [16]**	//	2 dietary patterns with a high intake of: VEG = vegetables, legumes, fruit, pasta fish and oil; PPP = pork, processed meat, potatoes, beef, rice, poultry, liver, butter/low-fat margarine, pasta and coffee	no significant association between VEG pattern and BC risk in any of the cohorts included in the analysis; in the multivariate model PPP was not significantly associated with BC risk in two cohorts (ORDET, SMC), while in the NLCS (Netherlands) cohort there was a significantly inverse association between PPP intake and BC risk (RR 0.90_0.81–0.99).	//	//	//
**Missmer 2002 [22]**	pre- and post-menopause	total meat, red and white meat, dairy fluids and solids	no significant association between total meat, red meat, white meat, total dairy fluids or solids and BC risk.	random effects model	pooled RR (highest vs. lowest quartile)	
					total meat 1.08 (0.98–1.19)	0.13
					red meat 0.94 (0.87–1.02)	0.13
					white meat 1.02 (0.91–1.13)	0.21
					dairy fluids 0.93 (0.84–1.03)	0.09
					dairy solids 1.01 (0.93–1.09)	0.94
**Smith-Warner 2001 (a) [46]**	pre- and post-menopause	saturated, monounsaturated, polyunsaturated, animal, vegetable and total fat intake	no statistically significant association between intake of fat subtypes and BC risk; no associations for animal or vegetable fat intake; these associations were unaffected by menopausal status.	random effects model	pooled adjusted RR (5% energy increases from specific fat subtypes):	
					saturated fat 1.09 (1.00–1.19)	
					- premenopause 1.10 (0.91–1.35)	0.61
					- postmenopause 1.07 (0.93–1.24)	0.61
					monounsaturated fat 0.93 (0.84–1.03)	
					- premenopause 0.87 (0.63–1.19)	0.46
					- postmenopause 0.81 (0.65–1.03)	0.46
					polyunsaturated fat 1.05 (0.96–1.16)	
					- premenopause 1.12 (0.88–1.41)	0.72
					- postmenopause 1.28 (0.96–1.69)	0.72
					animal fat 1.01 (0.96–1.06)	
					- premenopause 1.01 (0.91–1.12)	0.93
					- postmenopause 0.99 (0.95–1.03)	0.93
					vegetable fat 1.01 (0.98–1.04)	
					- premenopause 1.03 (0.93–1.13)	0.54
					- postmenopause 0.99 (0.94–1.04)	0.54
**Smith-Warner 2001 (b) [39]**	pre- and post-menopause	total fruits, fruit juice, total vegetables, total fruit and vegetables	weak, insignificant associations for total fruits, total vegetables, and total fruit and vegetables; no associations for green leafy vegetables, 8 botanical groups, and 17 specific fruits and vegetables; these associations were not influenced by menopausal status.	random effects model	pooled adjusted RR (highest vs. lowest quartile of intake):	
					total fruits 0.93 (0.86–1.00)	0.08
					total vegetables 0.96 (0.89–1.04)	0.54
					total fruits and vegetables 0.93 (0.86–1.00)	0.12
**Author_year**	**Menopausal status**	**Exposure measure**		**Overall results of review**		
**Results of systematic reviews included in the umbrella review (5)**						
**Albuquerque 2013 [17]**	//	dietary patterns (DP): - “Healthy/prudent”(fruits, vegetables, whole grains and cereals, fish and soy) -”Mediterranean”(fruit, raw and cooked vegetables, fish and crustaceans, olive and sunflower oil) - “Western” (red and processed meats, refined grains, potatoes and starches, snacks, sweets, fried food and soft drinks)		23 studies on the association of Healthy DP and BC risk (highest vs. lowest level of consumption):		
				- 12 studies found a significant inverse association with BC;		
				- 9 studies reported no statistically significant association;		
				- 2 case-control studies found a positive association.		
				3 studies assessed the Mediterranean DP (highest vs. lowest level of consumption):		
				- 2 studies found a significant inverse association with BC;		
				- 1 study report no statistically significant association.		
				24 studies examined the association of Western DP and BC risk (highest vs. lowest level of consumption):		
				- 9 studies found a positive association with BC;		
				- 14 studies reported no statistically significant association;		
				- 1 studies found a significant inverse association with BC.		
**Farsinejad-Marj 2015 [18]**	pre- and post-menopause for six studies; postmenopause for two studies	Mediterranean diet		4 studies considered the association between MD and BC risk in both pre- and post-menopausal women: two reported an inverse association; one found no association; two found an inverse association only in postmenopausal women; one found an inverse association only in premenopausal women. 2 studies considered the association between MD and BC risk only in postmenopausal women: 5. 1 found an inverse association; 6. 1 found no association.		
**Michels 2007 [28]**	pre- and post-menopause	dietary exposures: fat intake, fruit and vegetables consumption, antioxidants, vitamins (A, C, E, β-carotene), carbohydrates, glycemic index (GI) and glycemic load (GL), dairy food consumption (including vitamin D), soy and isoflavones, green tea, heterocyclic amines, adolescent diet		overall fat intake was unrelated to the incidence of BC; 2 cohorts showed a significant positive association between high fat intake and BC risk (ORDET cohort: RR 3.47_1.43–8.44);		
				fruit and vegetable consumption may prevent BC: only one significant association (Nurses’ Health Study) reported an inverse association for 5 or more vegetables/day;		
				vitamins A,C,E, beta-carotene: no consistent association (except for vitamin E intake in postmenopausal women);		
				carbohydrates, GI and GL: no significant association;		
				results for vitamin D, dairy and soy products were inconsistent.		
**Mourouti 2013 [34]**	//	soy consumption (isoflavones and lignans)		13 out of 16 case-control studies, 3 out of 3 nested case-control studies, and 3 out of 10 cohort studies demonstrated an inverse association between soy food, soy product or isoflavone intake and BC risk; some studies, 3 out of 16 case-control studies and 6 out of 10 cohort studies found no association between soy or isoflavone intake and BC risk; only 1 of 29 studies revealed a possible association between phytoestrogen dietary intake and BC risk.		
**Mourouti 2014 [21]**	pre- and post-menopause	intake of fruit and vegetables, meat, soy products and isoflavones, dietary fiber, dietary carbohydrates, dietary lipids		for fruit and vegetable consumption, most studies found a significant inverse association with BC risk;		
				for meat intake, 7 studies found a significantly higher risk and 7 found no association; most case-control studies on soy consumption found an inverse association;		
				most studies on dietary fiber found no association;		
				3 cohort and 2 case-control studies on dietary carbohydrates found a higher BC risk for higher GI and GL, while the other 11 studies found no association;		
				6 out of 10 case-control studies found a positive association with high fat intake, but most cohort studies found no significant association.		
**Results of qualitative reviews included in the umbrella review (7)**						
**Cui 2006 [54]**	pre- and post-menopause	dietary and supplemental vitamin D intake; dietary and supplemental calcium intake		7 studies (5 on pre- and post-menopausal women, 1 on postmenopausal women, 1 on premenopausal women) of the 12 studies considered found a statistically significant inverse association (highest vs. lowest category of intake) between calcium intake and BC risk; this study showed no association between dietary and supplemental vitamin D intake and BC; only one cohort study found a protective effect among premenopausal women.		
**Duffy 2007 [35]**	pre- and post-menopause	phytoestrogen intake		10 case-control studies showed a significant inverse association between phytoestrogen/isoflavone/lignin intake and BC risk, while 5 revealed no such association; only 1 of 6 cohort studies found a protective effect of high isoflavone intake.		
**Eichholzer 2001 [57]**	pre- and post-menopause	dietary folate intake		epidemiological studies suggested an inverse association between folate intake and BC among female regular alcohol consumers; no significant association between folate intake and BC risk in the overall population.		
**Hanf 2005 [23]**	//	fat intake; meat and fish consumption; fruit and vegetable consumption, fiber consumption		12 studies tested total fat intake as a risk factor for BC, and only one showed a statistically significant reduced RR with a higher total fat intake; animal fat was not found associated with BC; no significant association emerged for saturated, monounsaturated and polyunsaturated fatty acids: 4/10 prospective studies found a positive correlation between high levels of meat consumption and BC risk; no association between fish intake and BC; no significant correlation between BC and high consumption of fruit and vegetables; no overall association between fiber intake and BC risk: only 1 of 5 studies suggested an effect of fiber.		
**Lof 2006 [36]**	pre- and post-menopause	dietary lignan intake		4 of 5 case-control studies showed a protective effect of dietary lignans against BC, especially in premenopausal women; 2 prospective studies did not confirm any protective effect of dietary lignans.		
**Peeters 2003 [37]**	pre- and post-menopause	phytoestrogens (soybeans, soy products, miso, tofu, soy protein, isoflavones)		overall, results showed no protective effect of dietary intake of soy products against BC, except for women who consumed phytoestrogens in adolescence or in very high doses.		
**Rossi 2014 [60]**	//	proteins, carbohydrates and glycemic index (GI), dietary fat, polyphenols and phytoestrogens, fruits and vegetables, lycopene, vitamins and oligo elements, alcohol		consumption of well- or over-cooked red meat was associated with a higher risk of BC; n-3 PUFA may protect against BC; a lower GI seems associated with a lower risk of BC; high-fat diet, and high total cholesterol and triglyceride levels are associated with a higher risk of BC; evidence supports a protective role of lycopene, polyphenols, fruit and vegetables against BC; there was evidence of vitamin D reducing the BC risk; and of zinc and, to a lesser extent, vitamins E and B reducing the BC risk thanks to their antioxidant properties.		

**Table 3:** Conclusions and limitations of studies included in the umbrella review.

Author_ Year	Limitations	Recommendations/Conclusions	Quality Assessment Tool	Quality Assessment
**Conclusion and quality assessment of meta-analysis included in the umbrella review (32)**				
**Alexander 2010 [43]**	Meta-analysis limited to analysis of results reported across publications. Many studies on fat intake and BC probably did not examine fat from animal sources alone. Differential reporting of dietary factors may influence summary associations across the literature. The association between dietary fat and BC subgroups is also incomplete.	The available epidemiological evidence from prospective studies does not support an independent association between animal fat intake and BC risk. Better dietary assessments and methods in cohort studies, also examining dietary fat and BC in randomized clinical trials, may help to clarify any possible relationships.	//	//
**Aune 2012 [38]**	Potential unmeasured or residual confounders; a higher fiber intake is often associated with other lifestyle factors (higher levels of physical activity, lower prevalence of obesity, lower intake of alcohol and dietary fat); measurement errors in dietary intake assessments are known to bias effect estimates.	Diet with a high intake of plant-based foods rich in fiber could have an impact in the prevention of BC; this review suggests that diets rich in fiber are associated with a lower BC risk.	//	//
**Boyd 2003 [44]**	Measurement error in the food frequency questionnaires (FFQs) used in most studies may lead to overestimation of the range of intakes, and may also lead to attenuation of risk.	Combined risk estimates of for total and saturated fat intake, and for meat intake, all indicated an association between higher intakes and an increased risk of BC. Case-control and cohort studies gave similar results.	Quality of studies scored using preset methodological standards	Twenty-six studies defined as “higher-quality”
**Brennan 2010 [14]**	Possible recall bias in case-control combined analysis; possible weakness in design of the studies (different response rates and inconsistent adjustment for potential confounders). Possible misclassification within the two dietary patterns.	This review provided evidence of a small inverse association between a healthy diet and a positive trend between Western diet and BC risk. The results of this meta-analysis highlight the need for more carefully designed observational and intervention studies to clarify the influence of diet on BC risk.	//	//
**Buck 2010 [29]**	Potential measurement errors due to recall bias or shortcomings in food composition database.	High lignan exposure may be associated with a reduced risk of BC in postmenopausal women. Additional work is warranted to confirm this association.	//	//
**Chen 2010 [52]**	Not reported.	From the meta-analysis, there was a significant inverse association between vitamin D and calcium intake and BC risk; these findings support the use of vitamin D and calcium as chemopreventive agents against BC. Better designed clinical trials are needed to ascertain the protective effect and optimize the doses of these nutrients.	//	//
**Chen 2014 [55]**	The BC prevention effect of folate may be conferred by other nutrients in foods (leafy vegetables, legumes, egg yolk, baker’s yeast); dose–effect relationships for dietary folate intake level and BC risk differed between prospective and case-control studies; changing dietary habits during the follow-up in prospective studies was not assessed; differences in sample size, study region, study design and assessment methods may cause heterogeneity and prompt differences in the stratification analysis.	The findings revealed a potential non-linear dose effect for dietary folate levels against BC risk, and modest folate intake may reduce BC risk. Given the complexity of folate metabolism and uses, the dose and timing of folate intake should be considered. More prospective studies are necessary.	Newcastle–Ottawa Scale (NOS)	Thirty studies classified as “higher-quality” (≥5 points (pt)), twelve as “lower-quality” (<5 pt)
**Dong 2011 (a) [30]**	Strong degree of heterogeneity made results difficult to interpret. Changes in soy consumption during the long follow-up may have weakened the associations observed.	A statistically significant inverse association was confirmed between soy isoflavone intake and BC risk. The protective effect of soy against BC may be due to other, associated healthy lifestyle factors (high vegetable and fruit intake, more physical activity).	//	//
**Dong 2011 (b) [26]**	Consumption of dairy food, especially low-fat products, is probably associated with a healthy lifestyle; the likelihood of inadequate control for confounding factors may bias the findings; dietary assessments suffer from measurement error; possible misclassification bias; substantial heterogeneity across studies.	Findings indicate that a higher consumption of total dairy products may be associated with a reduced BC risk: menopausal status may influence this effect.	//	//
**Gandini 2000 [24]**	Possibility of publication bias (due to studies not being published if findings are not significant); heterogeneous intake categories.	This analysis confirmed the association between vegetable and, to a lesser extent, fruit intake and BC risk. Increasing vegetable consumption, and the intake of associated micronutrients (vitamin C and b-carotene) might reduce the risk of BC.	//	//
**Gissel 2008 [53]**	Few studies and low vitamin D intake reported in most trials; possible bias from diet reporting.	Increasing the intake of vitamin D above 400 IU/day may help to prevent BC; but more research is needed.	//	//
**Guo 2015 [19]**	The residual confounders in the studies are a problem in the meta-analysis of observational studies; these findings are probably affected by the misclassification of meat; the intake and consumption levels in the highest and lowest categories varied across studies; possibility of publication bias.	A high intake of red and/or processed meat was associated with an increased risk of BC. Additional cohort or interventional studies will be needed to confirm the association.	//	//
**Hu 2012 [59]**	Socio-economic position may influence the intake of carotenoids; the lack of original data in the studies limited the assessment of potential interactions; no cohort study in Asia focused on the associations between carotenoids and BC. This analysis did not assess the quality of all the studies included.	Total α-carotene intake could reduce the BC risk. The relationship between β-carotene intake and BC needs to be confirmed. No significant associations emerged between dietary β-cryptoxanthin, lutein+zeaxanthin, lycopene and BC.	//	//
**Hui 2013 [58]**	//	The study suggested that the intake of flavonols and flavones, but not of other flavonoid subclasses, is associated with a decreased risk of BC, especially after menopause.	//	//
**Larsson 2007 [56]**	Potential bias due to confounders inherent in the original studies; potential recall, interviewer or selection bias in case-control studies; heterogeneity due to methodological differences among the studies.	No clear support for an overall relationship between folate intake and BC risk. Adequate folate intake may have a protective effect against BC.	//	//
**Li 2014 [41]**	Relatively small number of eligible studies included in the analysis, and only two prospective studies; limited information extracted from the original studies; different types of mushroom.	The findings suggested that a higher edible mushroom consumption may be associated with a lower risk of BC; large-scale prospective studies are needed to confirm as much.	Newcastle–Ottawa Scale (NOS)—9 pt scoring system	Four studies classified as “higher-quality” (≥7 pt) three as “lower-quality” (<7 pt)
**Mulholland 2008 [47]**	Study results inconsistently adjusted for potential confounders might result in residual confounding elements; a relatively small number of studies included in the analysis, making it difficult to estimate publication bias and heterogeneity.	High dietary GI and GL do not appear to be of etiological importance in BC development.	Newcastle–Ottawa quality assessment Scale (NOS) —9 pt scoring system	Fourteen “higher-quality” studies
**Mullie 2016 [48]**	Not all studies reported women’s menopausal status. Lack of RR unadjusted and adjusted for adiposity.	The evidence supports a modest association between a dietary pattern with high GI/GL and the risk of BC.	//	//
**Schwingshackl 2014 [15]**	Relationship between diet and cancer is complex (multiple confounding variables); Mediterranean diet (MD) was not homogenous (heterogeneity on score items); possible measurement errors and recall/selection bias in the studies included.	No significant changes in risk reduction for BC by adherence to MD.	Newcastle–Ottawa quality assessment Scale (NOS)—9 pt scoring system	Eight “higher-quality” (≥7 pt) and three “lower-quality” studies (<7 pt)
**Seely 2005 [42]**	Small number of observational studies and no RCTs; marked variability among questionnaire response rates in cohort studies; unadjusted analysis for potential confounding factors; possible selection bias that could distort the findings; case-control studies produced weaker evidence than cohort studies.	This systematic review and meta-analysis found no significant effect of green tea on BC prevention. Green tea consumption may be associated with a reduced risk of BC recurrences.	//	//
**Si 2014 [25]**	Only articles written in English were included in the meta-analysis; possible selection bias; categorical standards for egg consumption were confused, making it difficult to them among the studies; cooking methods were not ascertained.	Pooled results of cohort studies and combined results showed that higher egg consumption was associated with a higher BC risk, especially for European and postmenopausal populations. More studies should be conducted using a well-recognize categorical standard for egg consumption. Egg consumption should be reduced to reduce BC risk.	//	//
**Song 2013 [40]**	Diversity of study design; the apparent association of these pooled results is restricted to the case-control study (overestimating the exposure effect due to recall and selection bias); measurement error in the FFQs; cut-off points for citrus fruit intake vary across studies, so there is uncertainty about the optimal citrus fruit intake for the prevention of BC.	Pooled results from observational studies showed an inverse association between citrus fruit intake and the risk of BC. This study underscores the need for well-designed prospective observational and intervention studies to clarify the influence of citrus fruit on BC.	//	//
**Taylor 2009 [20]**	Bias involving the use of FFQs in all studies (self-report measures to assess consumption can lead to misclassification of intake); recall bias in case-control studies may affect observed associations between dietary intake and cancer risk (tendency to over-report among cases and under-report among controls).	Red meat may be a source of heterocyclic amines and heme-iron, a highly bioavailable form of iron which has been shown to enhance estrogen-induced tumor formation. This quantitative summary of published literature indicates that red meat may contribute to BC risk in premenopausal women.	//	//
**Trock 2006 [31]**	Possible measurement errors among the studies; potential selection bias; different levels of soy consumption among the studies; potentially uncontrolled confounders among the studies.	Soy intake may be associated with a small reduction in BC risk. This result should be interpreted with caution, however, due to potential exposure misclassification, confounders, or lack of a dose-response effect.	//	//
**Turner 2011 [45]**	Small sample size in premenopausal studies; association between BC risk and PUFA subtypes not examined separately.	High-fat diet may increase estrogen levels and therefore increase BC risk. Higher risk of BC in postmenopausal women consuming large amounts of total fat and PUFA. Conversely, dietary fat may have some BC prevention effects in premenopausal women.	//	//
**Velentzis 2009 [32]**	Over- or under-estimation of food content; some food composition databases are incomplete; the amount of lignans in food can differ depending on crop variety, location, year of harvest and processing; possible dietary measurement errors associated with FFQs; possibility of residual confounding factors.	Plant lignans may be associated with a small (15%) reduction in postmenopausal BC risk, but further studies are needed to confirm these results.	//	//
**Wu 2008 [9]**	Not reported	Overall, data based on Asian women showed a dose-dependent statistically significant association between soy food intake and BC risk reduction; soy intake was unrelated to BC risk in studies conducted on Western populations.	//	//
**Wu 2015 [33]**	Different units used to assess food intakes among the studies; dose-response analysis was not conducted; possible measurement errors and analysis unadjusted for potential confounding factors.	High fruit and vegetable consumption, as a protective measure, can reduce the incidence of BC in Chinese women. High fruit consumption protects against BC (higher soy food consumption induces a 69% reduction in BC risk). High-fat food consumption may increase BC risk.	Newcastle–Ottawa quality assessment Scale (NOS)—9 pt scoring system	Eleven studies were “higher-quality” (≥7 pt) and eleven were “lower-quality” (<7 pt)
**Yang 2014 [49]**	Potential publication bias (small studies with null results tend not to be published); any measurement error and resulting misclassification would most likely lead to an attenuation of the true association; dietary questionnaires are liable to measurement errors (reporting bias or inaccurate databases); potential residual confounding factors not excluded.	Findings provide conclusive evidence to support increasing n-3/n-6 PUFA intake for BC prevention. For every 1/10 increment in n-3/n-6 PUFA there was a 6% reduction in BC risk; importance of promoting nutritional education (increasing the consumption of marine foods).	Newcastle–Ottawa quality assessment Scale (NOS)—9 pt scoring system	Six studies were classified as “high-quality” (≥8 pt), five as “moderate-quality” (<8 pt)
**Zang 2015 [27]**	Potential confounding factors inherent in the original study design; misclassification of dairy food consumption (self-reporting system) may cause incorrect estimates; potential methodological differences between studies; use of case-control studies may provide a lower level of evidence.	Dairy food consumption was inversely associated with BC risk; possible association between high dairy food consumption and beneficial environmental factors (economic status, physical activity, healthier lifestyle).	Newcastle–Ottawa quality assessment Scale (NOS)—9 pt scoring system	Twelve studies scored 8 pts, nine scored 7 pts, and six scored 6 pts
**Zheng 2013 [50]**	Different assessment methods; limited data available on individual n-3 PUFA; potential residual confounders not analyzed; possible language bias.	This meta-analysis provided strong and solid evidence of marine n-3 PUFA intake being inversely associated with risk of BC.	Newcastle–Ottawa quality assessment Scale (NOS)—9 pt scoring system	Studies were of “moderate-quality“ (5–7 pt) and “higher-quality” (>7 pt)
**Zhou 2015 [51]**	Selection bias might be unavoidable; relatively limited amount of data included in the meta-analysis; potential measurement errors and misclassification; unmeasured or residual confounders might affect the association between linoleic acid and BC risk	This meta-analysis suggested that linoleic acid is associated with a lower risk of BC, but none of the associations were statistically significant.	Newcastle–Ottawa quality assessment Scale (NOS)—9 pt scoring system	Four studies scored 6/9 pts, ten studies scored 7/9 pts
**Conclusion and quality assessment of pooled analysis included in the umbrella review (4)**				
**Mannisto 2005 [16]**	//	The results supported the suggestion deriving from relatively recent epidemiological research that diet may not have an important role in the etiology of BC. The possible protective effect of “Pork, Processed Meat, Potatoes” dietary pattern in the NLCS cohort could be explained by a difference in that pattern for NLCS (type of food included in the FFQs).	//	//
**Missmer 2002 [22]**	Inability to assess the effect of how food was cooked and for how long (data not collected); possible measurement errors not collected.	This large study does not provide evidence of a diet rich in meat and dairy products increasing the risk of BC.	//	//
**Smith-Warner 2001 (a) [46]**	Possible errors in estimates of consumption (over- or under-estimation); possible methodological errors in study design.	The pooled analysis suggested only a weak positive association for replacing saturated fat consumption with carbohydrate consumption; none of the other types of fat examined were associated with BC risk; these results, though not significant, are compatible with a lower BC risk observed for higher intakes of olive oil (rich source of monounsaturated fat) in studies conducted in Mediterranean countries.	//	//
**Smith-Warner 2001 (b) [39]**	Differences in study design; numerous fruit and vegetable categories reported in the studies; possible publication bias.	The results suggest that fruit and vegetable consumption in adulthood is not significantly associated with BC risk (BC risk only 3% to 9% lower in women in the highest decile of fruit or vegetable consumption). No specific fruits or vegetables had strong and significant association with BC risk.	//	//
**Conclusion and quality assessment of systematic reviews included in the umbrella review (5)**				
**Albuquerque 2013 [17]**	No summary estimate measure was produced; instruments used to collect dietary information differed among the studies; FFQs, used in most cases, are liable to measurement error and might not detect a significant association; great variability among the studies; possibility of recall or selection bias.	The results suggest that a dietary pattern characterized by vegetables, fruit, fish and soy (healthy), or the Mediterranean diet reduces the risk of BC. A positive association was observed for the Western dietary pattern, but most of these results were not statistically significant. Efforts are expected to aid future research.	Strobe Checklist	//
**Farsinejad-Marj 2015 [18]**	Scarcity of studies, no assessment of factors hypothesized to relate to BC (breastfeeding, adiposity, physical activity, genetic background, insulin resistance, and chronic inflammation). Few studies separately investigated risk of BC in pre- and post-menopausal women. Different components of the MD can act synergistically in reducing BC risk so it is important to consider all components of the MD in studies.	Case-cohort studies found an inverse association between the MD and BC risk in pre- and post-menopausal women, but cohort studies reported controversial results. It seems that there are insufficient data to reach a conclusion about the effect of MD on BC risk before and after menopause, but there is some evidence to suggest a protective association. More cohort studies need to be conducted in different parts of the world to confirm these results.	//	//
**Michels 2007 [28]**	Possible measurement error (FFQs); great heterogeneity in dietary patterns among different countries; timing in diet; possible inadequate follow-up in studies.	There was no consistent strong and statistically significant association between diet and BC risk.	//	//
**Mourouti 2013 [34]**	Possible recall bias; variation in exposure measures used in the studies; variation in the amount and type of soy consumed and in the sample size may have over- or under- estimated the true effect sizes measured. Potential dietary measurement error may mask the true relationship.	The majority of the studies (66%) revealed a statistically significant inverse association between soy and isoflavones consumption and BC risk. Limitations in the study design may mask the real associations.	//	//
**Mourouti 2014 [21]**	Differences in experimental design and habits among study populations; lack of sufficient follow-up in the studies; methodological problems with the assessment tools used in each study.	No consistent and statistically strong association emerged between BC incidence and dietary factors; soy food and isoflavone intake seemed to protect against BC, mainly in Asian populations; no association reported for the consumption of dietary carbohydrates, GI or GL, dietary fiber intake and the risk of BC. The consumption of dietary fat probably raises the risk of BC. The studies that examined the role of fruit, vegetable and meat intake provide inconsistent results.	//	//
**Conclusion and quality assessment of qualitative reviews included in the umbrella review (7)**				
**Cui 2006 [54]**	Possible selection bias due to case-control study design; shortcomings in methodology and summary estimate measures.	Despite inconsistent results from the epidemiological studies, some evidence indicates that vitamin D and calcium might have a protective role against BC. Experimental evidence suggests that the association between vitamin D intake and BC may be stronger for premenopausal than for postmenopausal women. Further analyses are required to assess this interaction.	//	//
**Duffy 2007 [35]**	Not reported	Early exposure in childhood or adolescence may have a protective effect against BC.	//	//
**Eichholzer 2001 [57]**	Not reported	High folate intake may be associated with a lower risk of BC in women at high risk (alcohol users).	//	//
**Hanf 2005 [23]**	Not reported	Regional differences in BC incidence are probably partially attributable to lifelong dietary habits.	//	//
**Lof 2006 [36]**	Potential misclassification of phytoestrogen intake due to measurement associated with dietary assessment; potential recall bias in retrospective studies.	Epidemiological data seem to support a small protective effect of isoflavones against BC; as for lignans and BC, the results are somewhat inconsistent, but the protective effect might be limited to premenopausal women.	//	//
**Peeters 2003 [37]**	None of the studies considered age at time of consumption, which seems to be important in BC development; methodological differences between the studies are too large to estimate any summary effect; limited number of epidemiological studies on phytoestrogens and BC.	None of the studies found evidence for an increased risk of BC with an increased phytoestrogen intake.	//	//
**Rossi 2014 [60]**	Limitations due to study design (methodological shortcomings and lack of association measures).	Nutritional factors might have a role in the development of BC; red meat intake and dietary sugar should be reduced, whereas increased fish intake appears to be protective; fruit and vegetables, including polyphenols, have shown promising results. Prospective randomized trials are needed to develop population-based prevention strategies for BC.	//	//

## Supplementary Materials

The following are available online at https://www.mdpi.com/1660-4601/17/13/4731/s1, Table S1: Summary of measures of BC risk associated with dietary exposures—For META-ANALYSES only; Table S2: Summary of measures of BC risk associated with dietary exposures; Table S3: Quality of studies included in the umbrella review.
