# Supplementary material for: Breast Cancer Primary Prevention and Diet: An Umbrella Review
Source: Int J Environ Res Public Health. 2020 Jul 1;17(13):4731. doi: 10.3390/ijerph17134731 (PMC7369836; doi:10.3390/ijerph17134731)
Supplement: Supplementary file 1 [file ijerph-17-04731-s001.pdf]

Table S1: Summary of measures of BC risk associated with dietary exposures. + significant positive association (>1); = no significant association; - inverse association (<1) –for META-ANALYSES only

**Type of food - Authors**	**Alexander 2010[43]**	**Aune 2012[37]**	**Boyd 2003[44]**	**Brennan 2010[14]**	**Buck 2010[28]**	**Chen 2010[52]**	**Chen 2014[55]**	**Dong 2011 (a)[29]**	**Dong 2011 (b)[25]**	**Gandini 2000[38]**	**Gissel 2008[53]**	**Guo 2015[19]**	**Hu 2012[59]**	**Hui 2013[58]**	**Larsson 2007[56]**	**Li 2014[41]**	**Mulholland 2008[47]**	**Mullie 2016[48]**	**Schwingshackl 2014[15]**	**Seely 2005[42]**	**Si 2014[24]**	**Song 2013[40]**	**Taylor 2009[20]**	**Trock 2006[30]**	**Turner 2011[45]**	**Velentzis 2009[31]**	**Wu 2008[9]**	**Wu 2015[32]**	**Yang 2014[49]**	**Zang 2015[26]**	**Zheng 2013[50]**	**Zhou 2015[51]**
**Dietary patterns**																																
Mediterranean diet																			=/- [i]													
Prudent/healthy diet				-/= [ii]																												
Western/unhealthy diet				=/+ [iii]																												
**Foods**																																
Dietary fiber, soluble and insoluble fiber		-																														
Fruits and fruit fiber		=								=																		-				
Vegetables and vegetable fiber		=								-																		-				
Cereal fiber		=																														
Soy (soy food, protein, flavonols, phytoestrogen, etc.)								-/= [iv]						-/= [v]										-			-/= [vi]	-				
Lignans					=/- [vii]																					=/- [viii]						
Citrus fruits																						-										

Green tea																				=/- [ix]												
Mushrooms																-																
Meat (total, red, processed)			+									+											+/= [x]									
Carbohydrates and food with HGI/HGL																	+	+/= [xi]														
Dairy food			=						-/= [xii]																					-/= [xiii]		
Eggs																					+/= [xiv]											
**Nutrients**																																
Vitamin C										-																						
Carotenoids										- [xv]			-/= [xvi]																			
Folate (total and dietary)							=/- [xvii]								=/- [xviii]																	
Fat (total fat, animal fat, saturated fat)	=		+																						=/+ [xix]			+				
MUFA			=																						=							
PUFA, n-3 PUFA, fish intake			=																						+				-/= [xx]		-/= [xxi]	=
Calcium						-/= [xxii]																										
Vitamin D											=/- [xxiii]																					

i. (highest vs. lowest categories of adherence to Mediterranean diet): = total studies, cohort studies; - case-control studies
ii. (highest vs. lowest categories of intake): -prudent/healthy overall, cohort studies combined; = case-control studies combined
iii. (highest vs. lowest categories of intake): = Western/unhealthy overall, cohort studies combined; + case-control studies combined
iv. among all women, those in Asian countries and those postmenopause; = among Western countries and premenopausal women

v. (highest vs. lowest categories of intake): - flavonol intake (overall and among postmenopausal women), flavone intake (overall and among postmenopausal women) and flavan-3-ol intake (among postmenopausal women only); = flavan-3-ol intake (overall), flavonone and anthocyanidin intake (overall)

vi. (highest vs. lowest categories of intake): - Asian women overall, pre- and postmenopausal Asian women; = Western women overall

vii. (highest vs. lowest quantile): = overall, and among premenopausal women (including dietary plant lignan intake category); - among postmenopausal women (including dietary plant lignan intake category)

viii. (highest vs. lowest categories of intake): = overall; - among postmenopausal women

ix. (highest vs. lowest consumption): = with random effect model (both cohort and case-control studies); - with fixed effect model (case-control studies only)

x. (highest vs. lowest categories of red meat intake): + overall and case-control studies; = cohort studies

xi. (highest vs. lowest categories of intake): + food with high GI/GL overall; = among both pre- and postmenopausal women

xii. (highest vs. lowest categories of intake): - total dairy food, overall and among premenopausal women; = milk, total dairy food among postmenopausal women, and total milk among both pre- and postmenopausal women)

xiii. (highest vs. lowest categories of intake): - cohort studies, overall and among premenopausal women, case-control studies; = cohort studies, among postmenopausal women

xiv. (highest vs. lowest categories of intake): + overall, cohort studies, among postmenopausal women, ≥2 and ≤5 eggs/week; = case-control studies, and among postmenopausal women

xv. β-carotenoids only

xvi. - α-carotenoids (cohort and case-control studies), β-carotenoids (cohort and case-control studies), lutein & zeaxanthin (case-control studies only), lycopene (case-control studies only); = β-cryptoxanthin (cohort and case-control studies), lutein & zeaxanthin (cohort studies only), lycopene (cohort studies only)

xvii. = total folate and dietary folate (prospective studies), total folate and dietary folate in premenopausal women (case-control studies); - dietary folate overall and in postmenopausal women (case-control studies)

xviii. (highest vs. lowest categories of dietary folate intake): = cohort studies; - case-control studies

xix. (highest vs. lowest quartile): = total fat, saturated fat and all fat types among premenopausal women; + all fat types among postmenopausal women

xx. (highest vs. lowest categories of n-3/n-6 PUFA intake): - overall and among postmenopausal women; = among premenopausal women

xxi. (highest vs. lowest categories of intake): - marine n-3 PUFA, overall and among postmenopausal women; = fish intake (overall and among pre- and postmenopausal women), and marine n-3 PUFA intake among premenopausal women

xxii. (highest vs. lowest categories of intake): - overall and among premenopausal women; = among postmenopausal women only

xxiii. = overall; - restricting analysis to highest (>400 IU/day) vs lowest intake

Table S2: Summary of measures of BC risk associated with dietary exposures. + significant positive association (>1); = no significant association; - inverse association (<1)

Type of study	POOLED ANALYSES				SYSTEMATIC REVIEWS					QUALITATIVE REVIEWS						
**Type of food - Authors**	Mannisto 2005[16]	Missmer 2002[22]	Smith-Warner 2001 (a)[46]	Smith-Warner (b)[39]	Albuquerque 2013[17]	M. Farsinejad-Marj 2015[18]	Michels 2007[27]	Mourouti 2013[33]	Mourouti 2014[21]	Cui 2006[54]	Duffy 2007[34]	Eichholzer 2001[57]	Hanf 2005[23]	Lof 2006[35]	Peeters 2003[36]	Rossi 2014[70]
**Dietary patterns**																
Mediterranean diet					-/= [i]	-/= [ii]										
Prudent/healthy diet	=				-/=/+ [iii]											
Western/unhealthy diet	=/- [iv]				-/=/+ [v]											
**Foods**																
Dietary fiber, soluble and insoluble fiber									-/=/+ [vi]				-/= [vii]			
Fruits and fruit fiber				-/= [viii]			=		-/= [ix]				-/= [x]			- [ix]
Vegetables and vegetable fiber							-/= [xii]		-/= [xiii]							- [xi]
Soy (soy food, protein, flavonols, phytoestrogen, etc.)							-/= [xiv]	-/=/+ [xv]	-/= [xvi]		-/= [xvii]				-/= [xviii]	- [xi]
Lignans														-/= [xix]		
Citrus fruits																
Green tea							=									
Mushrooms																
Meat (total, red, processed)		=							+/= [xx]				+/= [xxi]			+ [xi]
Carbohydrates and food with HGI/HGL							-/=/+ [xxii]		+/= [xxiii]							+ [xi]

Dairy foods		=					-/=/+ [xxiv]									
Eggs		+														
**Nutrients**																
Vitamins (A, B, C, E)							+/= [xxv]									- [xi]
Carotenoids							=									
Folate (total and dietary)												=				- [xi]
Total fat (animal fat, saturated fat)			+/= [xxvi]				-/=/+ [xxvii]		+/= [xxviii]				-/= [xxix]			+ [xi]
MUFA			=						+/= [xxx]				-/=/+ [xxxi]			
PUFA, n-3 PUFA, fish intake			=						+/= [xxxii]				-/=/+ [xxxiii]			- [xi]
Calcium										-/= [xxxiv]						- [xi]
Vitamin D										-/= [xxxv]						- [xi]

i. : - for 2 studies; = for 1 study
ii. : - for 2 studies on pre- and postmenopausal women, 3 on postmenopausal women, and 1 on premenopausal women; = for 1 study on both pre- and postmenopausal women, and 1 on postmenopausal women
iii. : - for 10 studies; = for 10 studies; + for 2 studies
iv. : (PPP: pork, processed meat and potatoes) = in ORDET and SMC cohorts; - in NLCS cohort
v. : - for 1 study; = for 15 studies; + for 8 studies
vi. : - 3 studies; = 5 studies; + 1 study
vii. : - for 1 study (borderline); = for 4 studies
viii. : - borderline for total fruits, total fruits & vegetables; = for total vegetables
ix. : - for 7 case-control studies; = for 2 case-control studies and 2 cohorts
x. : - for 1 study (among premenopausal women); = for 2 studies
xi. : Rossi analyzed 157 pertinent articles with the strongest level of evidence and provided no table of the results, making it hard to say how many studies showed some significance for single exposures, so the author's overall results are reported here.
xii. : - for 2 studies; = for 6 studies
xiii. : - for 5 case-control studies; = for 4 case-control studies and 2 cohorts
xiv. : - 1 study about pre- and postmenopausal women combined; 4 studies about postmenopausal women only;

xv. : - for 3 cohorts, 3 nested case-control studies and 13 case-control studies; = 6 cohorts and 3 case-control studies; + for 1 cohort
xvi. : - for 15 studies; = for 2 case-control studies, 6 cohorts and 1 nested case-control study
xvii. : - for 10 studies; = for 5 studies
xviii. : - for 4 case-control studies (high soy consumption, particularly among adolescents); = for 4 case-control studies and 4 prospective studies
xix. : - for 2 case-control studies (among premenopausal women) and 2 case-control studies (highest vs. lowest quartile); = for 2 prospective cohorts
xx. : + for 10 studies; = for 5 studies
xxi. : + for 4 studies; = for 6 studies
xxii. : - for 1 cohort; = 6 cohorts; + for 3 cohorts among postmenopausal women
xxiii. : + for 5 studies; = for 10 studies
xxiv. : - for 3 cohorts (all dairy foods, whole milk); = for 8 studies; + for 2 cohorts high-fat dairy foods, whole milk)
xxv. : + for 1 cohort (about vit. E); = for 10 cohorts
xxvi. : (increment of 5% of energy for each type of fat) + for saturated fat; = for animal fat and vegetal fat
xxvii. : - for 1 cohort among premenopausal and postmenopausal women combined; = for 18 studies; + for 2 cohorts among postmenopausal women
xxviii. : + for 10 studies about total fat and saturated fat; = for 11 studies about total fat and saturated fat
xxix. : - for 1 study about total fat and saturated fat; = for 12 studies about total fat, animal fat, saturated fat and plant fat
xxx. : + for 5 studies; = for 5 studies
xxxi. : - for 1 study; = for 6 studies; + for 2 studies (one of these is borderline)
xxxii. : + for 4 studies; = for 4 studies
xxxiii. : - for 1 study about fish intake; = for 7 studies about PUFA and for 6 studies about fish intake; + for 1 study about PUFA (borderline)
xxxiv. : - for 7 studies; = for 5 studies
xxxv. : - for 1 cohort; = for 7 studies

Table S3. Quality of studies included in the umbrella review.

	PRISMA 2009 - Checklist																											
Author/item	***1***	***2***	***3***	***4***	***5***	***6***	***7***	***8***	***9***	***10***	***11***	***12***	***13***	***14***	***15***	***16***	***17***	***18***	***19***	***20***	***21***	***22***	***23***	***24***	***25***	***26***	***27***	***Total***
Albuquerque 2013	1	0	1	1	0	1	1	1	1	1	1	1	0	0	0	0	1	1	1	1	0	0	0	1	1	1	0	17
Alexander 2010	1	0	1	1	0	1	1	0	1	1	1	0	1	1	1	1	0	1	0	1	1	1	1	1	1	1	1	21
Aune 2012	1	1	1	1	1	1	1	0	1	1	1	0	1	1	1	1	1	1	0	1	1	1	1	1	1	1	1	24
Boyd 2003	1	0	1	1	0	0	1	0	0	0	1	1	1	1	0	1	0	1	0	1	1	0	1	1	0	1	1	16
Brennan 2010	1	1	1	1	0	1	1	0	1	1	1	0	1	1	1	1	1	1	0	1	1	1	1	1	1	1	0	22
Buck 2010	1	1	1	1	0	1	1	0	1	0	1	0	1	1	1	1	0	1	0	1	1	1	1	1	1	1	0	20
Chen 2010	1	0	1	1	0	1	1	0	0	0	0	0	1	1	1	1	0	1	0	1	1	1	1	1	0	1	1	17
Chen 2014	1	1	1	1	1	1	1	0	1	1	1	1	1	1	1	1	1	0	0	1	1	1	1	1	0	1	1	23
Cui 2006	1	0	1	1	0	1	0	0	0	0	0	0	0	0	0	0	0	1	0	1	0	0	0	1	0	1	0	8
Dong 2011 (a)	1	1	1	1	0	1	1	0	1	0	1	0	1	1	1	1	0	1	0	1	1	1	1	1	1	1	0	20
Dong 2011 (b)	1	1	1	1	1	1	1	0	1	1	1	1	1	1	1	1	1	1	0	1	1	1	1	1	1	1	1	25
Duffy 2007	0	0	1	1	0	1	0	0	0	0	0	0	0	0	0	0	0	1	0	1	0	0	0	1	0	1	0	7
Eichholzer 2001	0	0	1	0	0	1	0	0	0	0	1	0	0	0	0	0	0	1	0	1	0	0	0	1	0	1	0	7
Farsinejad-Marj 2015	0	1	1	1	0	1	1	1	1	0	1	0	1	1	0	0	1	1	0	1	0	0	0	1	1	1	0	16

	PRISMA 2009 - Checklist																											
Author/item	***1***	***2***	***3***	***4***	***5***	***6***	***7***	***8***	***9***	***10***	***11***	***12***	***13***	***14***	***15***	***16***	***17***	***18***	***19***	***20***	***21***	***22***	***23***	***24***	***25***	***26***	***27***	***Total***
Gandini 2000	1	0	1	1	0	1	1	0	1	1	1	0	1	1	0	1	1	1	0	1	1	0	1	1	0	1	1	19
Gissel 2008	1	1	1	1	0	1	1	0	1	0	0	0	1	0	1	1	0	1	0	0	1	1	1	1	0	1	0	16
Guo 2015	1	1	1	0	0	1	1	1	1	1	1	1	1	1	1	1	1	1	0	1	1	1	1	1	1	1	1	24
Hanf 2005	0	0	1	1	0	0	0	0	0	0	1	0	0	0	0	0	0	1	0	1	0	0	0	1	0	1	0	7
Hu 2012	1	0	1	1	0	1	1	0	0	1	1	0	1	1	1	1	1	0	0	1	1	1	1	1	1	1	0	19
Hui 2013	1	1	1	1	0	1	1	0	1	0	1	0	1	1	1	1	0	1	0	1	1	1	1	1	0	1	1	20
Larsson 2007	1	1	1	1	0	1	1	0	1	0	1	0	1	1	1	1	1	1	0	1	1	1	1	1	1	1	0	21
Li 2014	1	1	1	1	0	1	1	0	1	1	0	1	1	1	1	1	1	1	0	1	1	1	1	1	1	1	1	23
Lof 2006	0	1	1	1	0	1	1	0	0	0	0	0	1	0	0	0	1	1	0	1	0	0	0	1	1	1	1	13
Mannisto 2005	0	1	1	1	0	0	0	0	1	0	1	0	1	1	0	1	0	1	0	1	0	0	1	1	0	1	1	14
Michels 2007	1	1	1	1	0	1	1	1	1	1	1	0	0	0	0	0	1	1	0	1	0	0	0	1	0	1	0	15
Missmer 2002	1	0	1	1	0	0	0	0	0	1	1	0	1	1	0	1	0	1	0	0	1	0	1	1	1	1	1	15
Mourouti 2013	0	0	1	1	1	1	1	0	1	0	0	0	0	0	0	0	1	1	0	1	1	0	0	1	1	1	1	14
Mourouti 2014	1	0	1	1	1	1	1	0	1	0	0	0	1	0	0	0	1	1	0	1	0	0	0	1	1	1	0	14
Mullie 2016	1	0	1	1	1	1	1	1	1	1	1	0	1	1	1	1	1	1	0	1	1	1	1	1	1	1	1	24
Mulholland 2008	1	0	1	1	0	1	1	0	1	1	1	1	1	1	1	1	1	1	0	1	1	1	1	1	1	1	1	23

	PRISMA 2009 - Checklist																											
Author/item	***1***	***2***	***3***	***4***	***5***	***6***	***7***	***8***	***9***	***10***	***11***	***12***	***13***	***14***	***15***	***16***	***17***	***18***	***19***	***20***	***21***	***22***	***23***	***24***	***25***	***26***	***27***	***Total***
Peeters 2003	1	0	1	1	0	1	0	0	1	0	0	0	1	0	0	0	1	1	0	1	0	0	0	1	0	1	1	12
Rossi 2014	0	1	1	1	0	1	0	0	0	0	0	0	0	0	0	0	1	1	0	1	0	0	0	1	0	1	0	9
Schwinshackl 2014	1	0	1	1	1	1	1	0	1	1	1	1	1	1	1	1	1	1	0	1	1	1	1	1	1	1	0	23
Si 2013	1	1	1	1	0	1	1	0	1	1	0	0	1	1	1	1	1	1	0	1	1	1	1	1	1	1	0	21
Seely 2005	1	1	1	1	0	0	1	0	1	1	1	0	1	1	1	1	1	1	0	1	1	1	1	1	1	1	1	22
Smith-Warner 2011 (a)	1	1	1	1	0	1	0	0	1	0	0	0	1	1	0	1	0	1	0	1	1	0	1	1	1	1	1	17
Smith-Warner 2011 (b)	1	0	1	1	0	1	0	0	0	1	0	0	1	1	0	1	0	1	0	1	1	0	1	1	1	1	1	16
Song 2013	1	0	1	1	0	1	1	1	1	1	1	0	1	1	1	0	1	1	0	1	1	1	0	1	1	1	0	20
Taylor 2009	0	0	1	1	0	1	1	0	0	0	0	0	0	0	0	0	0	1	0	1	1	0	0	1	1	1	0	10
Trock 2006	1	1	1	1	0	0	1	0	0	0	0	0	1	1	1	1	1	1	0	1	1	1	1	1	1	1	0	18
Turner 2011	1	0	1	1	0	0	1	0	0	0	1	0	1	0	0	1	1	1	0	0	1	0	1	1	1	1	0	14
Velentzis 2009	1	1	1	1	0	1	1	0	1	1	0	0	1	1	1	0	1	1	0	1	1	0	0	1	0	1	1	18
Wu 2008	0	0	1	1	0	1	0	0	0	1	0	0	0	0	0	0	0	1	0	1	1	0	1	1	0	1	1	11
Wu 2015	1	0	1	1	0	1	1	0	1	1	1	0	1	0	1	1	1	1	0	1	1	1	1	1	1	1	0	20
Yang 2014	1	1	1	1	1	1	1	0	1	1	1	0	1	1	1	1	1	1	0	1	1	1	1	1	1	1	1	24
Zang 2015	1	1	1	1	1	1	1	0	0	1	1	1	1	1	1	1	1	1	0	0	1	1	1	1	1	1	0	22

	PRISMA 2009 - Checklist																											
Author/item	***1***	***2***	***3***	***4***	***5***	***6***	***7***	***8***	***9***	***10***	***11***	***12***	***13***	***14***	***15***	***16***	***17***	***18***	***19***	***20***	***21***	***22***	***23***	***24***	***25***	***26***	***27***	***Total***
Zheng 2013	1	0	1	1	0	1	1	1	1	0	1	0	1	1	1	1	1	1	0	1	1	1	1	1	1	0	1	21
Zhou 2015	1	1	1	1	0	1	1	0	0	1	1	1	1	1	1	1	1	1	0	1	1	1	1	1	1	1	1	23
